# Effect of Sanitation on Soil-Transmitted Helminth Infection: Systematic Review and Meta-Analysis

**DOI:** 10.1371/journal.pmed.1001162

**Published:** 2012-01-24

**Authors:** Kathrin Ziegelbauer, Benjamin Speich, Daniel Mäusezahl, Robert Bos, Jennifer Keiser, Jürg Utzinger

**Affiliations:** 1 Department of Epidemiology and Public Health, Swiss Tropical and Public Health Institute, Basel, Switzerland; 2 University of Basel, Basel, Switzerland; 3 Department of Public Health and Environment, World Health Organization, Geneva, Switzerland; 4 Department of Medical Parasitology and Infection Biology, Swiss Tropical and Public Health Institute, Basel Switzerland; University of Otago, New Zealand

## Abstract

**Background:**

In countries of high endemicity of the soil-transmitted helminth parasites *Ascaris lumbricoides*, *Trichuris trichiura*, and hookworm, preventive chemotherapy (i.e., repeated administration of anthelmintic drugs to at-risk populations) is the main strategy to control morbidity. However, rapid reinfection of humans occurs after successful deworming, and therefore effective preventive measures are required to achieve public health goals with optimal efficiency and sustainability.

**Methods and Findings:**

We conducted a systematic review and meta-analysis to assess the effect of sanitation (i.e., access and use of facilities for the safe disposal of human urine and feces) on infection with soil-transmitted helminths. PubMed, Embase, ISI Web of Science, and the World Health Organization Library Database were searched without language restrictions and year of publication (search performed until December 31, 2010). Bibliographies of identified articles were hand-searched. All types of studies reporting data on sanitation availability (i.e., having access at own household or living in close proximity to sanitation facility), or usage, and soil-transmitted helminth infections at the individual level were considered. Reported odds ratios (ORs) of the protective effect of sanitation on soil-transmitted helminth infections were extracted from the papers or calculated from reported numbers. The quality of published studies was assessed with a panel of criteria developed by the authors. Random effects meta-analyses were used to account for observed heterogeneity. Thirty-six publications, consisting of 39 datasets, met our inclusion criteria. Availability of sanitation facilities was associated with significant protection against infection with soil-transmitted helminths (OR  =  0.46 to 0.58). Regarding the use of sanitation, ORs of 0.54 (95% confidence interval [CI] 0.28–1.02), 0.63 (95% CI 0.37–1.05), and 0.78 (95% CI 0.60–1.00) were determined for *T. trichiura*, hookworm, and *A. lumbricoides*, respectively. The overall ORs, combining sanitation availability and use, were 0.51 (95% CI 0.44–0.61) for the three soil-transmitted helminths combined, 0.54 (95% CI 0.43–0.69) for *A. lumbricoides*, 0.58 (95% CI 0.45–0.75) for *T. trichiura*, and 0.60 (95% CI 0.48–0.75) for hookworm.

**Conclusions:**

Despite a number of limitations (e.g., most studies used a cross-sectional design and were of low quality, with potential biases and considerable heterogeneity), our results reveal that sanitation is associated with a reduced risk of transmission of helminthiases to humans. Access to improved sanitation should be prioritized alongside preventive chemotherapy and health education to achieve a durable reduction of the burden of helminthiases.

*Please see later in the article for the Editors' Summary*

## Abstract 2

A systematic review and meta-analysis by Kathrin Ziegelbauer and colleagues finds that sanitation is associated with a reduced risk of transmission of helminthiases to humans.

## Editors' Summary

**Background:**

Worldwide, more than a billion people are infected with soil-transmitted helminths, parasitic worms that live in the human intestine (gut). Roundworm, whipworm, and hookworm infections mainly occur in tropical and subtropical regions and are most common in developing countries, where personal hygiene is poor, there is insufficient access to clean water, and sanitation (disposal of human feces and urine) is inadequate or absent. Because infected individuals excrete helminth eggs in their feces, in regions where people regularly defecate in the open, the soil becomes contaminated with eggs. People pick up roundworm or whipworm infections when they ingest these eggs after they have matured in the environment by eating raw, unwashed vegetables or by not washing their hands after handling contaminated soil (a common transmission route for children). In the case of hookworm, the immature, infective stages of the worms, which hatch in the soil, can penetrate human skin, and people usually become infected by walking barefoot on contaminated soil. Mild infections with soil-transmitted helminths rarely have symptoms, but severe infections can cause abdominal pain and diarrhea, weakness, and malnutrition that can impair physical and mental development. Many soil-transmitted helminth infections can be safely and effectively treated with anthelmintic drugs, but there is rapid reinfection after successful treatment.

**Why Was This Study Done?:**

In 2001, the World Health Organization endorsed preventative chemotherapy as the global strategy to control soil-transmitted helminthiasis. The key component of this strategy is regular administration of anthelmintic drugs to at-risk groups—children, women of childbearing age, and adults in high-risk occupations such as nightsoil reuse and farming. Although this strategy reduces illness caused by soil-transmitted helminths, it does not prevent rapid reinfection. To interrupt transmission and to achieve local elimination of helminthiasis, integrated control approaches that include access to sanitation and other complementary interventions of a primary prevention nature are needed. In this systematic review and meta-analysis, the researchers investigate whether the availability and/or use of sanitation facilities (latrines or toilets) lowers the risk of soil-transmitted helminth infections. A systematic review uses predefined criteria to identify all the research on a given topic; a meta-analysis is a statistical method that combines the results of several studies.

**What Did the Researchers Do and Find?:**

The researchers identified 36 publications that included data on sanitation availability and/or use and the number of people in the study population infected with one or more of three types of soil-transmitted helminths. Meta-analysis of the data from these publications indicates that, compared to people with no access to sanitation facilities, people with access to sanitation facilities were half as likely to be infected with soil-transmitted helminths. Specifically, the odds ratios (ORs; chances) of infection with soil-transmitted helminths among people with access to latrines compared to people without access to latrines were 0.46, 0.56, and 0.58 for roundworm, whipworm, and hookworm, respectively; for all three helminths combined, the OR was 0.49. Use of (as opposed to access to) sanitation facilities also protected against soil-transmitted helminth infection (ORs of 0.78, 0.54, and 0.63 for roundworm, whipworm, and hookworm infections, respectively). Finally, combining the data for both access and use, people who either had or used a latrine were half as likely to be infected with a soil-transmitted helminth as people who neither had or used a latrine (OR 0.51).

**What Do These Findings Mean?:**

The studies included in this systematic review and meta-analysis have several shortcomings. For example, most were cross-sectional surveys—studies that examined the effect of the availability/use of sanitation on helminth infections in a population at a single time point. Given this study design, people who had latrines may have shared other characteristics that were actually responsible for the observed reductions in the risk of soil-transmitted helminth infections. Moreover, the data on latrine availability and use was derived from questionnaires and may, therefore, be inaccurate because people are often ashamed to admit that they defecate outside. Finally, the overall quality of the included studies was low. Nevertheless, these findings confirm that providing access to, and promoting use of, sanitation facilities is an effective control measure for soil-transmitted helminthiasis. Thus, there should be more emphasis on expanding access to adequate sanitation in control strategies for soil-transmitted helminths. This change in emphasis would reinforce the effects of preventative chemotherapy and ongoing health education on helminthiasis, in an economic, sustainability, and public health sense. Importantly, it would also improve the control of other neglected tropical diseases such as schistosomiasis and trachoma and would reduce the incidence of diarrhea, and thus child mortality, in developing countries.

**Additional Information:**

Please access these websites via the online version of this summary at http://dx.doi.org/10.1371/journal.pmed.1001162.

- The US National Institute of Allergy and Infectious Disease provides information on infections caused by soil-transmitted helminths
- The US Centers for Disease Control and Prevention also provides detailed information on roundworm, whipworm, and hookworm infections
- The World Health Organization provides information on soil-transmitted helminths, including a description of the current control strategy; the Partners for Parasite Control newsletter Action Against Worms focuses on specific areas of worm control; a teacher's resource book entitled A Lively and Healthy Me that deals with educating children about worm infections is also available
- The Global Network for Neglected Tropical Diseases, an advocacy initiative dedicated to raising the awareness, political will, and funding necessary to control and eliminate the most common neglected tropical diseases, provides information on infections with roundworm (ascariasis), whipworm (trichuriasis), and hookworm
- Two international programs promoting water sanitation are the World Health Organization Water Sanitation and Health program and the World Health Organization/United Nations Childrens Fund Joint Monitoring Programme for Water Supply and Sanitation
- The Water Supply and Sanitation Collaborative Council and Practical Action have information about approaches and technologies for sanitation

## Introduction

An estimated 4.5 billion people are at risk of infection with one of the three common soil-transmitted helminths, namely, the roundworm (*Ascaris lumbricoides*), the whipworm (*Trichuris trichiura*), and the hookworms (*Ancylostoma duodenale* and *Necator americanus*) [1],[2]. Infection with soil-transmitted helminths is intimately connected with poverty, with the highest prevalence rates observed in low- and middle-income countries where hygiene is poor, access to safe, clean water is lacking, and sanitation is absent or inadequate [3]–[7]. More than 1 billion people are infected with one or multiple species of soil-transmitted helminths, and the global burden of disease owing to soil-transmitted helminthiases is estimated at 39 million disability-adjusted life years [2],[8]–[10]. Anemia and other morbidities (e.g., reduced physical and cognitive development) are the main reasons for this large global burden [4],[11],[12]. People are infected after ingesting eggs from contaminated soil or food (*A. lumbricoides* and *T. trichiura*), or through active penetration of the skin by infective larval stages present in contaminated soil (hookworm) [3]. Soil-transmitted helminths do not reproduce in the human host, and hence, each established helminth in the human body is a result of an infection event.

In 2001, the World Health Organization endorsed preventive chemotherapy as the global strategy to control morbidity due to soil-transmitted helminthiasis and schistosomiasis [9]. The key component of this strategy is to regularly administer safe and efficacious anthelmintic drugs to at-risk populations, with a target of reaching at least 75%, and up to 100%, of school-aged children [9],[13],[14]. While this strategy has a direct impact on morbidity, it does not prevent reinfection [15],[16], and it is recognized that complementary interventions are necessary to reduce the frequency of reinfection [16]–[19]. A large body of historic evidence [20]–[22] and recent experiences from China [23] suggest that integrated control approaches are essential for the interruption of transmission and local elimination of helminthiases. Improved access to sanitation is a key factor of integrated control programs [15]–[19],[24],[25].

We were interested in the evidence regarding sanitation (i.e., access to, and use of, facilities for the safe disposal of human urine and feces) and its effects on infection of humans with soil-transmitted helminths. A systematic review and meta-analysis were carried out to determine whether the availability and/or use of sanitation facilities was associated with a reduced risk of infection with soil-transmitted helminths from single or multiple species.

## Methods

### Search Strategy and Inclusion Criteria

We performed a systematic review and meta-analysis adhering to the MOOSE guidelines for reporting meta-analyses of observational studies (see Text S1) [26]. Our protocol is available in Text S2. In brief, we systematically searched PubMed, Embase, and ISI Web of Science, which are readily available and widely used electronic databases for systematic reviews in the health sciences. Additionally, the World Health Organization Library Database and the authors' own collections of articles were examined. Preliminary searches using the Cochrane Library and the CAB Abstracts revealed no additional studies, and hence these databases were not considered further. No restrictions on language or year of publication were made. Our search was performed until December 31, 2010. We employed a broad search using the following keywords: “sanitation,” “sanitary engineering,” “water supply,” and “waste management,” in combination with one of the following soil-transmitted helminth-related terms: “helminth,” “soil-transmitted helminth,” “geohelminth,” “ascaris,” “lumbricoides,” “trichuris,” “trichiura,” “hookworm,” “ancylostoma,” “duodenale,” “necator,” and “americanus.”

Additionally, two previous general reviews pertaining to water and sanitation and parasitic worm infections were examined for relevant references [27],[28]. The bibliographies of publications identified and deemed relevant were hand-searched for potential additional important articles. If an article was considered relevant, but data were not available in the format needed for our meta-analysis, the corresponding authors were contacted by E-mail and asked for supplementary information. All study types were eligible if they reported the prevalence (i.e., number of people infected among the examined population) of *A. lumbricoides*, *T. trichiura*, hookworm, or all three soil-transmitted helminths combined, stratified by the presence or absence of sanitation facilities or by the use or non-use of sanitation facilities. Since insufficient data were available to distinguish between different types of sanitation facilities, all types of latrines (e.g., pit latrines, ventilated improved pit latrines, and flush toilets) were pooled. Hence, studies reporting only the presence or absence of latrines without further specificity regarding the type of latrines were eligible for inclusion. Open defecation was defined as no sanitation. Studies that only compared the effect of different toilet types (e.g., flush toilet *versus* pit latrine) were excluded. Regarding the use of sanitation, we also applied a broad set of inclusion criteria. For instance, studies that employed a questionnaire and asked one of the following questions “do you use a sanitary facility?” or “where do you defecate?” were included.

However, most intervention studies were excluded, because of specific aspects of the design, setting, and the complexity of interventions (e.g., multiple control measures) where the studies were implemented. Indeed, it is difficult to compare intervention studies carried out over different time frames and to distinguish studies that used single or multiple interventions (sanitation plus water supply, preventive chemotherapy, and health education) [29].

### Data Extraction and Quality Assessment

In the first step, studies identified in our computer-aided search that failed to meet at least one inclusion criterion after scrutinizing the title and, if available, the abstract, were excluded. In the second step, two reviewers (K. Z. and B. S.) independently examined the full text of potentially relevant articles using a standard protocol developed by the authors (see Text S2). In case of disagreement, a third reviewer (J. K. or J. U.) independently examined such articles, and the assessors' findings were discussed until consensus was reached.

Relevant data, including a brief description of the study (e.g., study design, setting, year, and sample size), the primary research question pursued by the study, details of the study population (e.g., all age groups, school-aged children only, or other special groups) and the selection of study population (e.g., random selection), specificities on sanitation facilities (i.e., availability or use), and the helminth species investigated were extracted from all eligible studies by K. Z. using a standard protocol and independently cross-checked by B. S.

The reported odds ratios (ORs) served as effect measures. For studies that did not report ORs, these were calculated from 2×2 contingency tables of sanitation facility (availability or use) and infection status with soil-transmitted helminths, compared to the infection status of those who do not have access to, or use, sanitation facilities. Whenever possible, reported ORs were used; if both adjusted and unadjusted ORs were reported, we considered unadjusted ORs. Studies reporting effect measures for more than one helminth species were considered, and relevant results were fed into the respective meta-analyses.

Inspired by the GRADE methodology [30], we developed a panel of criteria to assess the quality of identified studies. Our criteria focused on parasitological/diagnostic features, sanitation, and overall strengths and limitations of the studies. With regard to parasitological/diagnostic features, a study was given one point if the diagnostic approach (clinical assay) was clearly spelled out. Studies that employed a rigorous diagnostic approach (i.e., multiple stool samples examined and/or concurrent use of several diagnostic tests) received one additional point. Finally, studies that detailed an approach for quality control (e.g., 10% of stool examinations checked by a senior laboratory technician) were further given one additional point. Of note, no qualitative ranking of the different diagnostic tests was performed, as the sensitivity and specificity of a particular test depends on the overall endemicity (prevalence and intensity) of soil-transmitted helminthiasis. Conversely, studies that did not mention clinical/diagnostic assays were given zero points. With regard to sanitation-related quality assessment, a study was given one point if the toilet status (e.g., cleanliness and condition of superstructure) was investigated by the research team. Repeated spot checks of random sub-samples of sanitation availability and use were deemed sufficient to obtain a point. However, no point was assigned if the toilet status was assessed using a questionnaire, as questionnaires were not considered sufficient to be awarded a quality point. Finally, studies were scrutinized for other strengths (+1 point) and limitations (−1 point) (e.g., no random population sample, but instead high-risk group only). Two assessors (K. Z. and B. S.) performed the quality assessment independently and documented the results in separate tables. Results were discussed; in case of discrepancies, a third reviewer (J. K. or J. U.) examined the respective articles, and the ratings were discussed until consensus was reached among the assessors. Overall, a study could obtain an overall score ranging between −1 and +6 points. Since these ratings are mainly to inform the reader about the overall quality of individual studies, no studies were excluded because of low quality.

All studies were pooled in the meta-analyses and stratified by soil-transmitted helminth species (overall OR). Furthermore, we carried out separate meta-analyses for *A. lumbricoides*, *T. trichiura*, hookworm, and soil-transmitted helminths combined, stratified by (i) availability or use of sanitation facility; (ii) data for children, adults, or all age groups; and (iii) geographical area (Africa, Asia, South and Central America, and the United States).

### Statistical Analysis

ORs were calculated for specific soil-transmitted helminths by comparing prevalence rates among those individuals having access to, or using, sanitation and those without, or not using, facilities employing the “metan” code of Stata version 10 (StataCorp). StatsDirect version 2.4.5 (StatsDirect) was used for meta-analyses, performed for *A. lumbricoides*, *T. trichiura*, hookworm, and soil-transmitted helminths combined. Egger's test was utilized to investigate whether there was a publication bias (a small study bias is evident if *p*<0.1) [31]. Heterogeneity between studies was determined using Moran's *I* ^2^ and Cochran's *Q*-tests. Factors specified a priori as potential explanations for observed heterogeneity were age and type of toilet. Since there was some evidence for heterogeneity (*I* ^2^>50%), random effects models [32] were used throughout, and pooled ORs for the effect of sanitation on the prevalence of helminth infections were employed. Studies with an OR less than 1.0 indicate a decrease in the odds of being infected with soil-transmitted helminths among those individuals having access or using sanitation facilities.

## Results

### Inclusion, Exclusion, and Yielded Studies

**Figure 1:**
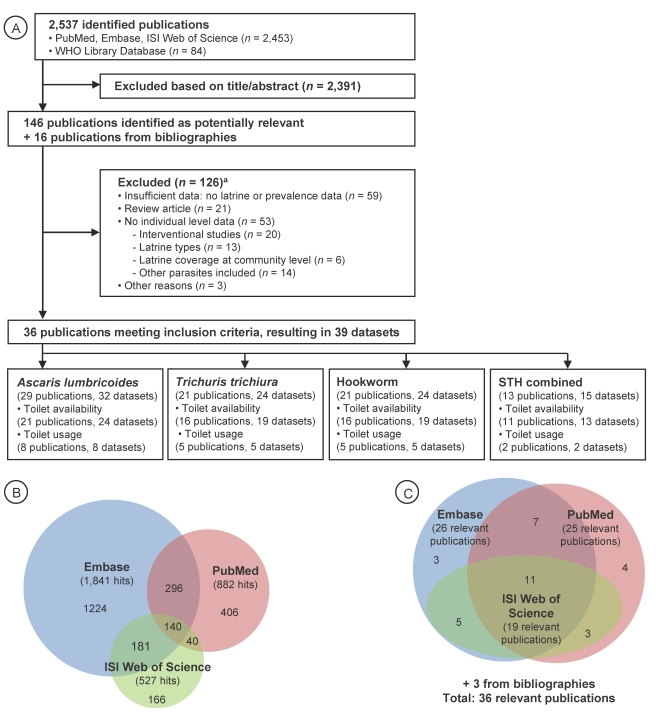
Flowchart visualizing the procedure for identifying relevant publications. Overall, 36 publications were identified, containing 39 datasets (A). Number of hits (B) and ultimate identification of relevant publications (C) are also shown, for three different electronic databases. STH, soil-transmitted helminths. ^a^Multiple exclusion criteria possible.

Our computer-aided search yielded 2,537 publications (Figure 1A), with the majority retrieved by Embase (1,841 hits) and PubMed (882 hits) (Figure 1B). From the titles and, when available, the abstracts of these articles, 146 publications were deemed relevant, hence, were fully screened by two of us (K. Z. and B. S.). The majority of relevant articles were obtained from Embase and PubMed (Figure 1C). Bibliographies of these 146 articles revealed an additional 16 studies that were also investigated by the first two authors. We noted missing data to address our research question in 34 publications, and, hence, the corresponding authors were contacted by E-mail. We received the requested data from ten authors pertaining to 12 studies, which were included in our analyses. Table S1 provides a summary of the 162 fully screened publications, including the reasons why studies were excluded. Thirty-six studies met our inclusion criteria—consisting of 39 datasets that were finally included in our meta-analyses—investigating the relationship between sanitation facilities and prevalence of soil-transmitted helminth infections.

Twenty-five publications investigated the effect of sanitation availability on infection with soil-transmitted helminths, whereas the remaining 11 articles focused on the use of sanitation and infection with soil-transmitted helminths. From the 36 publications, 16 focused on Asia [33]–[48], 11 on Africa [49]–[59], four on Central America [60]–[63], four on South America [64]–[67], and one on the United States [68]. The study conducted in the United States was the oldest one identified (published in 1970). With the exception of one article published in Spanish [66] and one in Chinese [38], articles were published in English. There were only two studies that reported results on intensity of soil-transmitted helminth infection, as determined by the number of helminth eggs per gram of stool [37],[69].

Of note, multiple studies dating back to the early decades of the last century from the southern part of the United States, Panama, and elsewhere also reported an impact of sanitation (often in combination with chemotherapy and other control measures) on soil-transmitted helminth infections [20],[22],[70]–[72]. However, these studies did not report data in the format needed for the current meta-analysis, and it was not possible to contact the authors by E-mail; hence, these studies were not considered further (see Table S1).

### Study Characteristics and Data Quality

Most of the publications identified were descriptive cross-sectional surveys, assessing single or multiple risk factors for infection with soil-transmitted helminths (Table 1). Only one intervention study was included in our meta-analysis, and this study was included because complete baseline data were available [49]. In 16 publications it was possible to obtain relevant data in a 2×2 contingency table format directly from the respective articles. The ten authors who kindly supplied the requested supplementary data for 12 studies upon E-mail inquiry did this in the form of 2×2 contingency tables as per our request. In five studies, the ORs provided in the articles were retrieved and used for subsequent meta-analysis. In three surveys, data were reanalyzed to obtain the respective contingency table information for meta-analyses. Study participants were chosen at random, either at individual or at household level in more than half of the relevant studies. In 14 studies, all individuals of a particular community, village, or special population group were enrolled, whereas no selection criteria for study participation were specified in four studies.

**Table 1:** Characteristics of studies examining the association of sanitation availability or sanitation use with soil-transmitted helminth infections, including quality assessment.

Reference	Study Design and Setting	Year	Study Population (Selection)	Availability (A) or Use (U) of Sanitation	Soil-Transmitted Helminth Species	Data Obtained	Diagnostic Approach (D)		Sanitation (S)		Other Strengths and Limitations (O)	Points	
							Method	Quality Control	Toilet Status Assessment Method	Spot Checks		D/S/O	Total
Al-Mekhlafi et al. [33]	Descriptive study in one school in Malaysia	2006	Sc (random)	A	A.l.	OR (MVA)	K-K (Hw: H-M)	n.s.	Questionnaire	n.s.	—	+1/0/0	+1
Asaolu et al. [52]	Descriptive study in two communities in Nigeria	1998	PSc (all)	A	A.l.	2×2 table	Mod. K-K	n.s.	Questionnaire	No	—	+1/0/0	+1
Basualdo et al. [64]	Descriptive study in one town of Argentina	2002	All age groups (n.s.)	A	A.l.; T.t.	2×2 table^a^	Mod. T-L (five samples)	n.s.	n.s.	n.s.	—	+2/0/0	+2
Belo et al. [53]	Descriptive study in three schools in Sao Tomé	2000	Sc (random)	U	A.l.; T.t.	OR (UVA)	K-K; T-L	n.s.	Questionnaire	n.s.	—	+2/0/0	+2
Chongsuvivatwong et al. [41]	Descriptive study in four villages in Thailand	n.s.	All >6 y (random HH)	U	Hw	2×2 table	K-K	n.s.	Questionnaire	Yes	Spent 11 mo prior to study to establish good relationship	+1/+1/+1	+3
Corrales et al. [61]	Case-control study in eight communities in El Salvador	n.s.	All age groups (random HH; all solar latrine owners)	A	A.l.; T.t.; Hw	OR (MVA)	Mod. RFEC	n.s.	n.s.	n.s.	—	+1/0/0	+1
de Souza et al. [65]	Descriptive study in two villages in Brazil	2004	All age groups (all)	A	STH; A.l.; T.t.; Hw	2×2 table^a^	SS	No	Questionnaire	No	—	+1/0/0	+1
Ensink et al. [34]	Descriptive study in four communities in Pakistan	2002	Adult men and children (only textile laborers, wastewater farmers, farmers)	A	STH; A.l.; T.t.; Hw	2×2 table^a^	FES	n.s.	n.s.	n.s.	Only high-risk groups	+1/0/−1	0
Erlanger et al. [46]	Descriptive study in 17 villages in Lao People's Democratic Republic	2001/2002	All age groups (random)	A	STH; A.l.; T.t.; Hw	2×2 table^a^	FES	n.s.	Questionnaire	n.s.	—	+1/0/0	+1
Gloor et al. [68]	Descriptive study in eight schools in the US	1968	Sc (all)	A	STH; Hw	2×2 table	ZSF	n.s.	Questionnaire	n.s.	—	+1/0/0	+1
Gunawardena et al. [35]	Descriptive study in one village in Sri Lanka	2000	All >2 y (random HH, participants)	A	A.l.; T.t.^a^	2×2 table^a^	K-K	n.s.	Questionnaire	n.s.	—	+1/0/0	+1
Gunawardena et al. [36]	Descriptive study in two plantations in Sri Lanka	2000	All age groups (n.s.)	A	Hw	2×2 table^a^	K-K	n.s.	Questionnaire	No	—	+1/0/0	+1
Hagel et al. [67]	Descriptive study in an urban slum in Venezuela	1993	Children (representing overall socio-economic structure)	A	A.l.; T.t.	2×2 table	Stoll	n.s.	Prior door-to-door interviews	n.s.	—	+1/0/0	+1
Holland et al. [60]	Descriptive study in one health center in Panama	1983	PSc (random)	A	A.l.; T.t.; Hw	2×2 table	n.s.	n.s.	Questionnaire (with mother or caregiver of child)	n.s.	—	+1/0/0	+1
Ilechukwu et al. [59]	Descriptive study in three nurseries and three schools in Nigeria	2003	PSc, Sc (random)	U	STH; A.l.; T.t.; Hw	2×2 table	K-K	n.s.	Questionnaire	n.s.	—	+1/0/0	+1
Jombo et al. [54]	Descriptive study in three communities in Nigeria	2004	All age groups (random)	A	STH; A.l.; T.t.; Hw	2×2 table^a^	Mod. DS	n.s.	Questionnaire	n.s.	—	+1/0/0	+1
Kightlinger et al. [50]	Descriptive study in southeast Madagascar	n.s.	Children (n.s.)	U	A.l.	2×2 table	FES	n.s.	Questionnaire	n.s.	—	+1/0/0	+1
Knopp et al. [58]	Descriptive study in two communities in Zanzibar	2008	All age groups (all adults; first 100 children)	A	STH; A.l.; T.t.; Hw	2×2 table^a^	K-K, BM, KAP	10% of stool samples	Questionnaire	n.s.	—	+3/0/0	+3
Matthys et al. [55]	Descriptive study in six communities in Côte d'Ivoire	2004	All age groups (all farmers; non-farmers: random)	U	Hw	2×2 table^a^	FEC, K-K (two slides)	10% of stool samples	Questionnaire	n.s.	—	+3/0/0	+3
Morales-Espinoza et al. [63]	Descriptive study in 32 communities in Mexico	1998	Children (systematic)	A	A.l.	OR (UVA)	Faust (three samples)	n.s.	Questionnaire	n.s.	—	+2/0/0	+2
Nguyen et al. [47]	Descriptive study among women of reproductive age in Viet Nam	1995	Women (random cluster sampling)	U	A.l.; T.t.; Hw	2×2 table	K-K	n.s.	Questionnaire	n.s.	Only data from closed latrine vs. “bush,” since open latrine not clearly defined	+1/0/0	+1
Nishiura et al. [37]	Descriptive study in five schools in Pakistan	2000	Sc (random)	U	A.l.	2×2 table	K-K (one stool)	n.s.	Questionnaire	n.s.	—	+1/0/0	+1
Olsen et al. [51]	Descriptive study in three villages in Kenya	1994	All age groups (all >4 y)	A	A.l.; Hw	2×2 table	K-K (two stools, two slides)	n.s.	Questionnaire	n.s.	—	+2/0/0	+2
Raja'a et al. [43]	Descriptive study in one town in Yemen	n.s.	Sc (random)	U	STH	2×2 table	Mod. K-K	n.s.	Questionnaire	No	—	+1/0/0	+1
Steinmann et al. [48]	Descriptive study in 51 schools in Kyrgyzstan	2009	Sc (random)	U	A.l.	Cal.	K-K (two slides)	n.s.	Questionnaire	n.s.	Toilet use during the night	+1/0/−1	0
Stephenson et al. [49]	Intervention study in two villages in Kenya	1975–1980	Sc, PSc (all)	A	A.l.	Cal.	Mod. T-L	n.s.	Questionnaire	n.s.	—	+1/0/0	+1
Stothard et al. [56]	Descriptive study in ten villages in Zanzibar	2006	Mothers and children (n.s.)	A	STH; A.l.; T.t.; Hw	2×2 table^a^	K-K (one slide)	10% of stool samples	Questionnaire	n.s.	—	+2/0/0	+2
Sun et al. [38]	Descriptive study in three counties in China	2003	All age groups (random)	A	STH	2×2 table	Mod. K-K	n.s.	n.s.	n.s.	—	+1/0/0	+1
Toma et al. [39]	Descriptive study in four villages in Indonesia	1994	All age groups (random HH)	A	A.l.; T.t.; Hw	2×2 table	Mod. K-K (Hw: mod. H-M)	n.s.	Questionnaire	No	—	+1/0/0	+1
Torres et al. [66]	Descriptive study in six schools in Chile	1993	Sc (all)	A	A.l.; T.t.	2×2 table	PAFS	n.s.	Questionnaire	n.s.	—	+1/0/0	+1
Trang et al. [45]	Descriptive study in two communities in Viet Nam	2003	Adults (random HH; exclusion of farmers)	A	STH; A.l.; T.t.; Hw	2×2 table^a^	DS	n.s.	Questionnaire	n.s.	—	+1/0/0	+1
Trang et al. [44]	Descriptive study in a peri-urban area in Viet Nam	2002	All age groups (random HH)	A	STH; A.l.; T.t.; Hw	2×2 table^a^	DS (one stool)	n.s.	Questionnaire	n.s.	—	+1/0/0	+1
Traub et al. [40]	Descriptive study in three communities in India	2000	All age groups (random HH)	U	A.l.; T.t.; Hw	Cal.	SS (one stool)	n.s.	Questionnaire	n.s.	Always the same interviewer	+1/0/+1	+2
Ugbomoiko et al. [57]	Descriptive study in one village in Nigeria	2005	Children (random HH)	A	A.l.	2×2 table	K-K (one sample)	n.s.	Questionnaire	n.s.	—	+1/0/0	+1
Wördemann et al. [62]	Descriptive study in two municipalities (19 schools) in Cuba	2003	Sc (all)	U	A.l.; T.t.; Hw	OR (UVA)	DS, K-K (two slides)	n.s.	Questionnaire	n.s.	—	+2/0/0	+2
Yajima et al. [42]	Descriptive study in one community in Viet Nam	2007	All age groups (random participant)	A	A.l.; T.t.; Hw	2×2 table	K-K (two slides)	n.s.	Questionnaire	n.s.	Small sample size (only three with no latrine)	+1/0/−1	0

a Data provided by author.

A.l., *Ascaris lumbricoides*; BM, Baermann (technique); Cal., calculated; DS, direct smear; FEC, formalin-ether concentration (technique); FES, formalin-ether sedimentation (technique); HH, household; H-M, Harada-Mori (technique); Hw, hookworm; KAP, Koga agar plate (technique); K-K, Kato-Katz (technique); Mod., modified; MVA, multivariate analysis; PAFS, polyvinyl alcohol fixative solution; PSc, pre-school children; n.s., not stated; RFEC, Ritchie's formalin-ether concentration (technique); Sc, schoolchildren; SS, stool sedimentation (technique); STH, soil-transmitted helminths; T-L, Teleman-Lima (technique); T.t., *Trichuris trichiura*; UVA, univariate analysis; ZSF, zinc sulfate flotation (technique).

The diagnostic technique utilized for assessing soil-transmitted helminth infection status was mentioned in all the studies meeting our inclusion criteria. The Kato-Katz technique [73] was the most widely used diagnostic approach (*n*  =  20). Three studies mentioned that quality control for microscopic examination of stool samples was performed. Only one study explicitly stated that repeated spot checks for sanitation facilities were done per protocol by the researchers [41].

Table 1 also summarizes the overall quality of the included studies. On our scale from −1 (worst quality) to +6 (best quality), most studies had a score of +1 (*n*  =  23) and, hence, were of relatively low quality. Quality of three studies was even lower (zero points), whereas the remaining ten studies had a score of +2 (*n*  =  7) or +3 (*n*  =  3). Two of the studies with the highest score pursued a rigorous diagnostic approach for detecting infections with soil-transmitted helminths (i.e., multiple stool samples, different techniques employed, and quality control) [55],[58]. One study had such a small sample size (i.e., only three persons without latrine), that one quality point was subtracted [42].

### Effect of Sanitation Availability and Use on Infections with Soil-Transmitted Helminths

**Figure 2:**
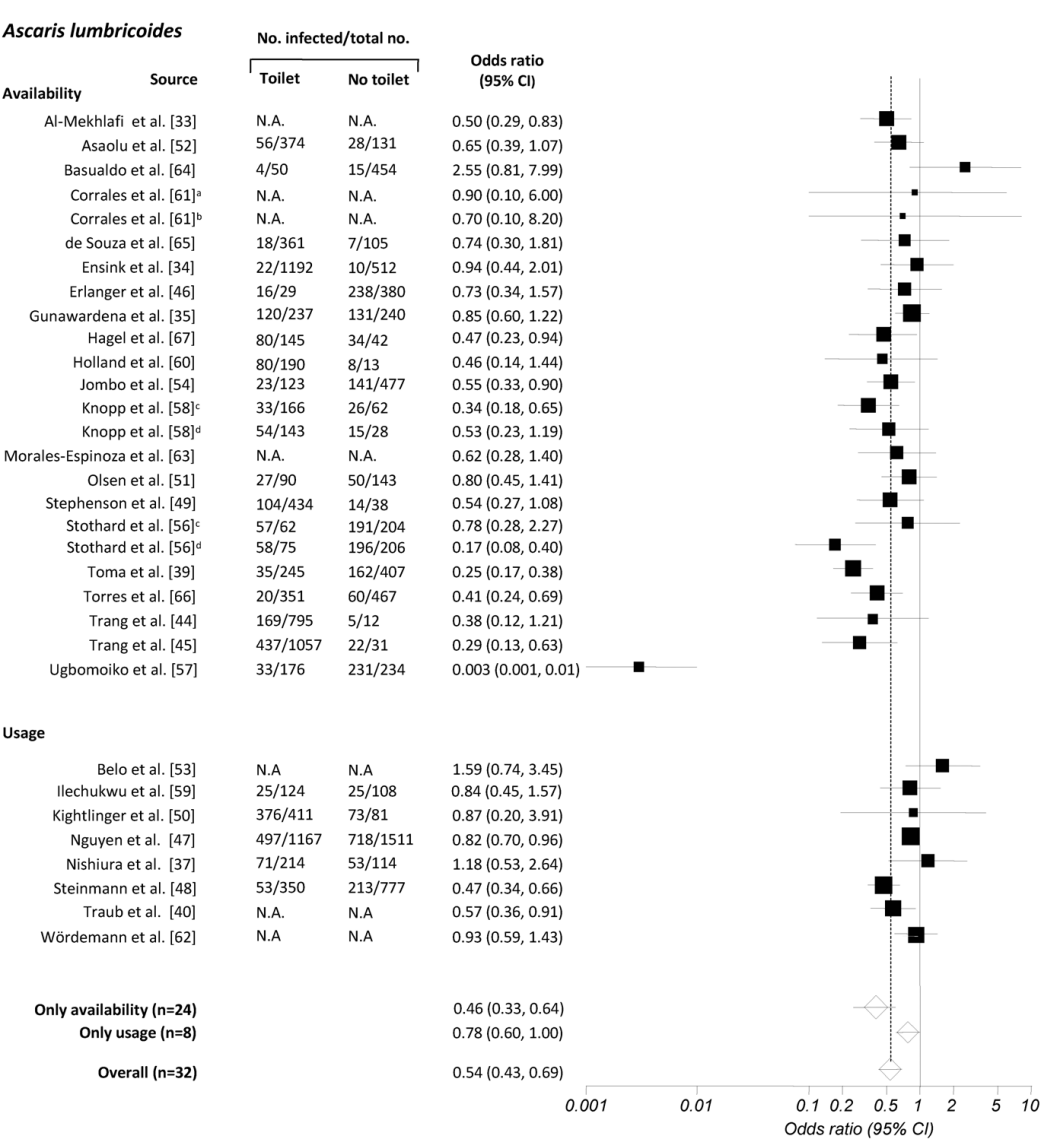
Meta-analysis examining the association of sanitation facilities with *A. lumbricoides* infection. Data are presented separately for availability and use of sanitation. Rectangles indicate ORs, and sizes of the rectangles represent the weight given to each study in the meta-analysis; open diamonds and vertical dashed lines indicate combined ORs; and horizontal lines indicate 95% CIs. Data are presented separately for ^a^only pit latrine, ^b^only solar urine-diverting desiccating latrine, ^c^only adults, ^d^only children. N.A., not assessed.

**Figure 3 pmed-1001162-g003:**
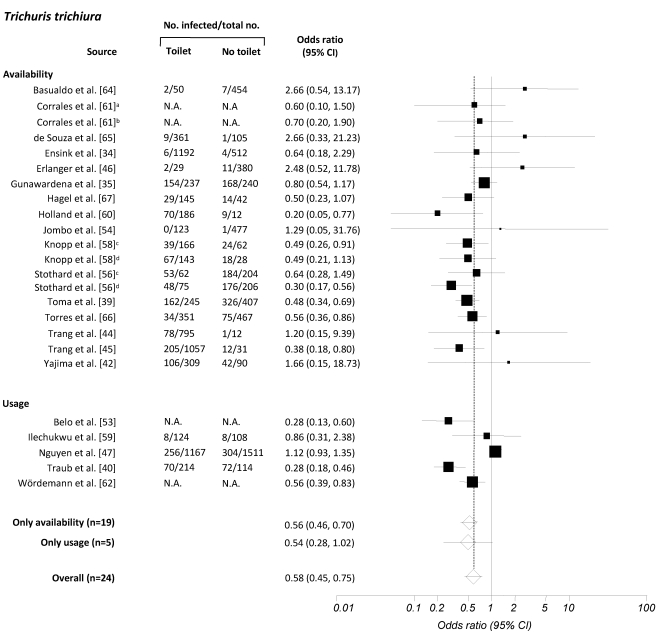
Meta-analysis examining the association of sanitation facilities with *T. trichiura* infection. Data are presented separately for availability and use of sanitation. Rectangles indicate ORs, and sizes of the rectangles represent the weight given to each study in the meta-analysis; open diamonds and vertical dashed lines indicate combined ORs; and horizontal lines indicate 95% CIs. Data are presented separately for ^a^only pit latrine, ^b^only solar urine-diverting desiccating latrine, ^c^only adults, ^d^only children. N.A., not assessed.

**Figure 4 pmed-1001162-g004:**
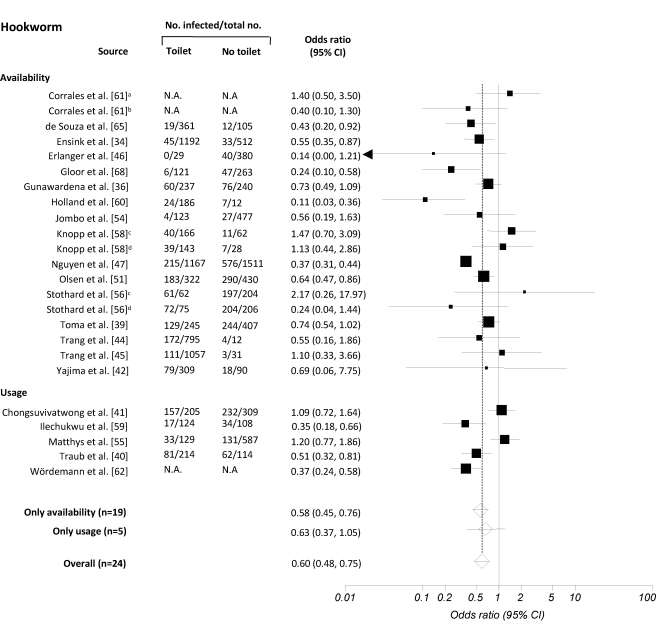
Meta-analysis examining the association of sanitation facilities with hookworm infection. Data are presented separately for availability and use of sanitation. Rectangles indicate ORs, and sizes of the rectangles represent the weight given to each study in the meta-analysis; open diamonds and vertical dashed lines indicate combined ORs; and horizontal lines indicate 95% CIs. Data are presented separately for ^a^only pit latrine, ^b^only solar urine-diverting desiccating latrine, ^c^only adults, ^d^only children. N.A., not assessed.

**Figure 5:**
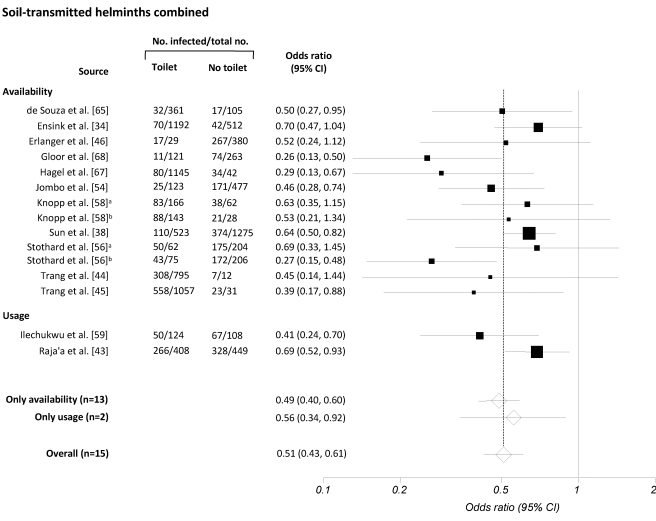
Meta-analysis examining the association of sanitation facilities with infection with the three common soil-transmitted helminths combined. Data are presented separately for availability and use of sanitation. Rectangles indicate ORs, and sizes of the rectangles represent the weight given to each study in the meta-analysis; open diamonds and vertical dashed lines indicate combined ORs; and horizontal lines indicate 95% CIs. Data are presented separately for ^a^only adults and ^b^only children.

Figures 2–5 present the effect estimates of sanitation availability and use for *A. lumbricoides* (Figure 2), *T. trichiura* (Figure 3), hookworm (Figure 4), and soil-transmitted helminths combined (Figure 5). The observed heterogeneity for the different sub-group meta-analyses, *I* ^2^, ranged from 0% (e.g., soil-transmitted helminths combined for studies conducted in Asia, and *T. trichiura* for studies carried out in Africa) to 90.5% (*A. lumbricoides*, sanitation use for studies carried out in Africa), justifying the use of random effects models for all meta-analyses (Table 2).

**Table 2:** Summary results of sub-group analysis examining the association of sanitation with soil-transmitted helminth infections.

Characteristics	*A. lumbricoides*			*T. trichiura*			Hookworm			Soil-Transmitted Helminths Combined		
	*n*	Random Effects Pooled OR (95% CI)	*I* ^2^ (%)	*n*	Random Effects Pooled OR (95% CI)	*I* ^2^ (%)	*n*	Random Effects Pooled OR (95% CI)	*I* ^2^ (%)	*n*	Random Effects Pooled OR (95% CI)	*I* ^2^ (%)
Overall	32	0.54 (0.43, 0.69)	80.7	24	0.58 (0.45, 0.75)	69.4	24	0.60 (0.48, 0.75)	71.0	15	0.51 (0.44, 0.61)	35.5
Only availability	24	0.46 (0.33, 0.64)	81.2	19	0.56 (0.46, 0.70)	20.5	19	0.58 (0.45, 0.76)	65.8	13	0.49 (0.40, 0.60)	33.3
Only use	8	0.78 (0.60, 1.00)	56.1	5	0.54 (0.28, 1.02)	90.5	5	0.63 (0.37, 1.05)	79.1	2	0.56 (0.34, 0.92)	N.A.
All age groups	16	0.61 (0.43, 0.80)	68.2	16	0.69 (0.49, 0.98)	71.5	18	0.70 (0.54, 0.90)	71.8	9	0.60 (0.51, 0.70)	0.0
Only children	16	0.46 (0.30, 0.71)	86.0	8	0.47 (0.37, 0.60)	14.3	6	0.35 (0.21, 0.57)	51.5	6	0.39 (0.26, 0.59)	66.7
Africa	12	0.41 (0.22, 0.77)	89.0	7	0.44 (0.32, 0.59)	0	8	0.77 (0.51, 1.17)	60.0	6	0.46 (0.35, 0.60)	14.7
Asia	11	0.57 (0.43, 0.77)	77.3	9	0.66 (0.41, 1.05)	82.7	10	0.62 (0.45, 0.86)	74.4	6	0.64 (0.55, 0.75)	0
Central and South America	9	0.67 (0.48, 0.96)	34.3	8	0.58 (0.43, 0.79)	14.3	5	0.42 (0.22, 0.78)	62.3	2	0.41 (0.24, 0.69)	N.A.
US	0			0			1	0.24 (0.10, 0.58)	N.A.	1	0.26 (0.13, 0.50)	N.A.

N.A., not assessed.

The 36 publications identified included 32 datasets on the effect of sanitation on infection with *A. lumbricoides*, 24 on infection with *T. trichiura*, 24 on infection with hookworm, and 15 on infection with all three soil-transmitted helminths combined. The estimated pooled random effects ORs of either having or using sanitation facilities compared to those individuals who neither have nor use a latrine were 0.54 (95% confidence interval [CI] 0.43–0.69) for infection with *A. lumbricoides*, 0.58 (95% CI 0.45–0.75) for *T. trichiura*, 0.60 (95% CI 0.48–0.75) for hookworm, and 0.51 (95% CI 0.43–0.61) for infection with soil-transmitted helminths combined.

Twenty-eight datasets were identified that specifically examined the relationship between availability of sanitation facilities and the prevalence of infection with soil-transmitted helminths. Among these, 24 reported data on *A. lumbricoides*, 19 on *T. trichiura*, 19 on hookworm, and 13 on soil-transmitted helminths combined. Although we observed wide ranges in effectiveness estimates, most studies showed that having access to a sanitation facility reduces the odds of being infected with soil-transmitted helminths, regardless of the species. The highest protective effect was observed for *A. lumbricoides* and soil-transmitted helminths combined, with respective summary estimates of 0.46 (95% CI 0.33–0.64; Figure 2) and 0.49 (95% CI 0.40–0.60; Figure 5). For infection with *T. trichiura* or hookworm, ORs of 0.56 (95% CI 0.46–0.70) and 0.58 (95% CI 0.45–0.76), respectively, were found (Figures 3 and 4). Evidence for publication bias was found for infection with soil-transmitted helminths combined pertaining to usage and availability of sanitation (*p*  =  0.017). We found a borderline significance for publication bias for sanitation availability alone (*p*  =  0.054). All other meta-analyses revealed no evidence of publication bias (Egger's test, *p*>0.1).

Use of sanitation facilities was reported in 11 publications. Stratified by soil-transmitted helminth species, meta-analyses included eight studies for *A. lumbricoides* (Figure 2), five for *T. trichiura* (Figure 3), and five for hookworm (Figure 4). Only two publications reported the relationship between use of sanitation facilities and infection with soil-transmitted helminths combined (OR  =  0.56; 95% CI 0.34–0.92). In the comparison of individuals who use a latrine with those who do not, the odds of being infected with *A. lumbricoides*, *T. trichiura*, and hookworm were 0.78 (95% CI 0.60–1.00), 0.54 (95% CI 0.28–1.02), and 0.63 (95% CI 0.37–1.05), respectively.

Results from different sub-group analyses are summarized in Table 2. The pooled OR of datasets examining only children (including pre-school and school-aged children [aged below 16 y]) ranged from 0.35 (95% CI 0.21–0.57) for infection with hookworm to 0.47 (95% CI 0.37–0.60) for infection with *T. trichiura*, suggesting a stronger association of sanitation with helminth infection in children than in the whole population. However, 95% CIs are strongly overlapping. Analyses of studies conducted in different geographical areas (Africa, Asia, South and Central America, and the United States) revealed no difference in associations between availability or use of sanitation facilities and infection with any of the common soil-transmitted helminth species.

## Discussion

Since the International Drinking Water Supply and Sanitation Decade (1980–1990), adequate sanitation, safe drinking water, and appropriate hygiene have been forgotten pillars of health, until recently [18],[19],[74],[75]. Fortunately, though, interest in access to safe, clean drinking water and adequate sanitation and improved hygiene has been renewed, and a road map of what needs to be done has been established [75]. Indeed, the United Nation's Millennium Development Goal 7c aims at halving the proportion of the population without sustainable access to safe drinking water and basic sanitation by 2015 [76], and the United Nation's General Assembly recently adopted access to water and sanitation as a basic human right [77]. Progress toward Millennium Development Goal 7c and recognizing water and sanitation as a basic human right will undoubtedly result in major health gains and improved well-being, such as lower incidence of diarrheal episodes and infant mortality, and enhanced human dignity, apart from other benefits [18],[75].

In our meta-analysis we found that the availability and use of sanitation facilities were associated with a reduction in the prevalence of infection with soil-transmitted helminths. Considering all of the studies that met our inclusion criteria, summary ORs ranging between 0.54 and 0.60 for the three common soil-transmitted helminth species were found. Similar estimates were obtained when studies were stratified by availability (ORs between 0.46 and 0.58) *versus* use of sanitation facilities (ORs between 0.54 and 0.78). Sub-group analysis, with stratification according to geographical area or children *versus* all age groups, showed no differences.

Our findings revealed a somewhat stronger negative association of lack of sanitation with infection with soil-transmitted helminths than previous general reviews in which the introduction of water supply and/or sanitation interventions was associated with a reduction in the prevalences of *A. lumbricoides* and hookworm of only 29% and 4%, respectively [27],[28]. These previous reviews included only one and four intervention studies for *A. lumbricoides*, and both identified only one relevant study for hookworm [78]. Interestingly, these earlier general reviews did not identify estimates for the association of sanitation with infection with *T. trichiura* and soil-transmitted helminth infections combined.

### Strengths and Limitations

We adhered to the MOOSE guidelines for reporting meta-analysis of observational studies (see Text S2) and performed electronic searches on three readily available and widely used databases (i.e., PubMed, Embase, and ISI Web of Science), supplemented with hand-searches of bibliographies of relevant articles and other sources, until December 31, 2010. We assessed and graded the quality of included studies (see Table 1). However, a number of shortcomings must be highlighted. First, the majority of studies identified reported only on prevalence of infections with soil-transmitted helminths rather than intensity, although the latter measure is of key relevance for morbidity. Indeed, only two of the identified studies assessed the effect of sanitation on infection intensity of soil-transmitted helminths, and hence, no meta-analysis could be performed. Second, we focused on individual-level data. We were therefore not able to address how intervention coverage and use in a community would modify the effect on the individual. It is conceivable that the health effect of changes in intervention coverage in a community from, say, 10% to 70% is distinctively different for the individual living in that community than if coverage increased from 70% to 100%. Unfortunately, this kind of data could not be extracted from the final set of studies included in our meta-analysis. The change in coverage and use of sanitation facilities between the time of baseline and follow-up is a potentially important determinant of impact and a potential explanation of heterogeneity. Third, we noted a publication bias regarding the results of all three soil-transmitted helminth species combined. However, Egger's tests on the individual helminth species did not indicate any publication bias, and hence, the reported ORs for the soil-transmitted helminths combined seem to be justified. Fourth, we did not include “grey literature” or expert consultations. Although this might have yielded important additional studies, we felt that standardization would have been too complicated and, hence, might have introduced additional biases.

Another aspect worth mentioning is that availability, access, ownership, and use of sanitation facilities are not one and the same. Indeed, availability of sanitation facilities does not automatically mean that people use them [43]. Therefore, we stratified results into availability and use of sanitation facilities in our meta-analysis. Our results do not suggest that use of sanitation facilities is more strongly associated than availability with infection by soil-transmitted helminths. This finding is not surprising, since one of the methodological shortcomings of our analysis is that studies reporting on the availability and use of sanitation facilities were both included. Availability and use of sanitation facilities was primarily assessed by questionnaires rather than verified by random spot checks or direct observations. It is conceivable that the question “Where do you defecate?” is prone to reporting bias, as people might be ashamed to state that they practice open defecation [79]. Moreover, farmers, fishermen, street vendors, and traders might have sanitation facilities at home and use them, but may be forced to practice open defecation or defecate in unimproved latrines (open pits) with highly contaminated surroundings during extended periods away from home. In view of this, one study focusing on school-aged children was excluded because the authors examined the availability of sanitation facilities only at school, and not at home [80].

Finally, in most of the included studies the type of sanitation facilities available or used was not mentioned, but such information is important, as the types of sanitation might be differentially associated with the prevalence of infection with different soil-transmitted helminth species [81]. If the type of sanitation facilities was mentioned, a wide variety of terms was used (e.g., flush toilet, water closet, ventilated improved pit latrine, pit latrine, and open latrine). Hence, there is a need for a more unified classification of latrine types. The “sanitation ladder” proposed by the World Health Organization/United Nations Children's Fund Joint Monitoring Programme for Water Supply and Sanitation is a first step in this direction [82]. In the current study, however, stratified analysis according to toilet type was not possible because of the lack of data. Other determinants that were not investigated in our meta-analysis were coverage levels of toilet availability and toilet use in a community, and the maintenance of sanitation facilities. Proper maintenance of toilets is crucial, as otherwise sanitation facilities can turn into “hookworm-traps” [83],[84]. Coverage plays an important role; only a few individuals defecating openly can maintain the transmission of helminths [85]. In addition, a recent study carried out in Viet Nam found high prevalence of soil-transmitted helminth infections despite the fact that 98.1% of the households owned a latrine. This was explained by the use of “night soil” (human excreta) as fertilizer, which is a common agricultural practice in many Asian countries [42].

There were no randomized controlled trials evaluating the impact of sanitation facilities on the prevalence of infection with soil-transmitted helminths identified in our systematic review. Although randomized controlled trials provide the most robust evidence [86], this experimental design is not always feasible, as seen in the current review and in other environmental interventions that have been tested to reduce the burden of infectious diseases [28],[87]–[91]. Intervention studies have the disadvantage that in addition to sanitation, more complex interventions were implemented, including health education, improvement of water supplies, and preventive chemotherapy. Obviously, it is then the package of interventions and not just one component that is associated with the outcomes [89],[91]. Furthermore, most studies have only short evaluation periods, and it is difficult to draw inferences regarding sustainability [92],[93]. It is interesting to note that only a few such complex integrated interventions were identified for sanitation and prevalence of helminth infections, and all except one were excluded. In cross-sectional observation studies, which make up most of our included studies, sanitation facilities had been in place for several years, and hence, the long-term effect on soil-transmitted helminth infections could be assessed. However, observational studies bear the risk of confounding, since people owning sanitation facilities may be different from those without. For example, community members owning and using sanitation facilities may be wealthier, their educational level might be higher, or they might be more health conscious [94].

### Policy Implications

The results of our meta-analysis reveal that sanitation is associated with a reduction in the prevalence of soil-transmitted helminth infections. Our findings, therefore, underscore what the Rockefeller Sanitary Commission stated more than 70 years ago: “Cure alone is almost useless in stamping out hookworm disease, because the patient can go out and immediately pick up more hookworms. The cure should be accompanied by a sanitation campaign for the prevention of soil pollution” [6]. Implementation of sanitation facilities and integrated control approaches go far beyond the prevention and control of intestinal helminths; they impact other neglected tropical diseases, such as schistosomiasis, trachoma, and diarrhea [23]–[25],[95], and can even help promote social and educational advances for women and girls [96]. For a durable impact, the process of implementing improved sanitation requires community involvement and setting-specific information, education, and communication strategies as key factors to ultimately change human behaviors. Now that the elimination of neglected tropical diseases is coming to the forefront of global attention, integrated control approaches—using a combination of preventive chemotherapy; information, education, and communication campaigns; and improvements to basic sanitation and access to safe, clean water—cannot be emphasized enough.

## Supporting Information

Table S1 Details of all the publications that were fully screened by the first two authors (n  =  162). Reasons why studies have been excluded are given (n  =  126). Studies included in our metaanalysis are shaded grey (n  =  36).
(DOC)

Text S1 PRISMA checklist.
(DOC)

Text S2 Study protocol for systematic review and meta-analysis to determine the effect of sanitation on soil-transmitted helminth infection.
(DOC)

## Abbreviations

CI: confidence interval
OR: odds ratio
