# Supplementary material for: Effect of Sanitation on Soil-Transmitted Helminth Infection: Systematic Review and Meta-Analysis
Source: PLoS Med. 2012 Jan 24;9(1):e1001162. doi: 10.1371/journal.pmed.1001162 (PMC3265535; doi:10.1371/journal.pmed.1001162)
Supplement: Table S1 — Details of all the publications that were fully screened by the first two authors (n = 162). Reasons why studies have been excluded are given (n = 126). Studies included in our metaanalysis are shaded grey (n = 36). (DOC) [file pmed.1001162.s001.doc]

**Table S1** Details of all the publications that were deemed relevant, and hence, fully screened by the first two authors (n=162). Reasons why studies have been excluded are given (n=126). Studies included in our meta-analysis are marked with light grey color (n=36).

| Reference (author, year) [reference no.] | Inclusion (I), exclusion (E) | Reasons for exclusion (0=no, 1=yes) | | | | | | |  |
| --- | --- | --- | --- | --- | --- | --- | --- | --- | --- |
|  |  | Review | Other parasites included | Latrine type | Latrine coverage | Multiple interventions | Insufficient data | No data at individual level | Other reasons |
| Abu Mourad (2004) [S1] | E | 0 | 1 | 0 | 0 | 0 | 0 | 1 | 0 |
| Acka et al. (2010) [S2] | E | 0 | 0 | 0 | 0 | 0 | 1 | 0 | 0 |
| Adeoye et al. (2007) [S3] | E | 0 | 0 | 1 | 0 | 0 | 0 | 1 | 0 |
| Aimpun and Hshieh (2004) [S4] | E | 0 | 0 | 0 | 0 | 0 | 1 | 0 | 0 |
| Albonico et al. (2006) [S5] | E | 1 | 0 | 0 | 0 | 0 | 0 | 0 | 0 |
| Al-Mekhlafi et al. (2007) [33] | I | 0 | 0 | 0 | 0 | 0 | 0 | 0 | 0 |
| Amuta et al. (2010) [S6] | E | 0 | 0 | 0 | 0 | 0 | 1 | 0 | 0 |
| Anantaphruti (2000) [S7] | E | 0 | 0 | 0 | 0 | 0 | 1 | 0 | 0 |
| Anthony et al. (2009) [S8] | E | 0 | 0 | 0 | 0 | 0 | 1 | 0 | 0 |
| Antunes et al. (2009) [S9] | E | 0 | 0 | 0 | 0 | 0 | 1 | 0 | 0 |
| Arfaa et al. (1977) [78] | E | 0 | 0 | 0 | 0 | 1 | 0 | 1 | 0 |
| Asaolu and Ofoezie (2003) [28] | E | 1 | 0 | 0 | 0 | 0 | 0 | 0 | 0 |
| Asaolu et al. (2002) [52] | I | 0 | 0 | 0 | 0 | 0 | 0 | 0 | 0 |
| Barreto et al. (2010) [S10] | E | 0 | 0 | 0 | 0 | 1 | 0 | 1 | 0 |
| Bartlett (2003) [S11] | E | 1 | 0 | 0 | 0 | 0 | 0 | 0 | 0 |
| Basualdo et al. (2007) [64] | I | 0 | 0 | 0 | 0 | 0 | 0 | 0 | 0 |
| Bath et al. (2010) [S12] | E | 0 | 0 | 0 | 0 | 0 | 1 | 0 | 0 |
| Bdir and Adwan (2010) [S13] | E | 0 | 0 | 0 | 0 | 0 | 1 | 0 | 0 |
| Belo et al. (2005) [53] | I | 0 | 0 | 0 | 0 | 0 | 0 | 0 | 0 |
| Belyhun et al. (2010) [S14] | E | 0 | 0 | 0 | 0 | 0 | 1 | 0 | 0 |
| Blum and Feachem (1983) [S15] | E | 1 | 0 | 0 | 0 | 0 | 0 | 0 | 0 |
| Boia et al. (1999) [S16] | E | 0 | 0 | 0 | 0 | 0 | 1 | 0 | 0 |
| Borda et al. (1996) [S17] | E | 0 | 0 | 0 | 0 | 0 | 1 | 0 | 0 |
| Bradley et al. (1993) [S18] | E | 0 | 0 | 0 | 0 | 1 | 0 | 1 | 0 |
| Cairncross (1987) [81] | E | 1 | 0 | 0 | 0 | 0 | 0 | 0 | 0 |
| Cairncross (1989) [S19] | E | 1 | 0 | 0 | 0 | 0 | 0 | 0 | 0 |
| Cairncross (2003) [S20] | E | 1 | 0 | 0 | 0 | 0 | 0 | 0 | 0 |
| Cairncross et al. (2010) [88] | E | 1 | 1 | 0 | 0 | 0 | 0 | 1 | 0 |
| Carneiro et al. (2002) [69] | E | 0 | 0 | 1 | 0 | 0 | 0 | 1 | 0 |
| Silva (2007) [S21] | E | 0 | 0 | 0 | 0 | 0 | 1 | 0 | 0 |
| Chandiwana et al. (1989) [S22] | E | 0 | 0 | 0 | 0 | 0 | 1 | 0 | 0 |
| Chandler (1954) [S23] | E | 0 | 0 | 0 | 0 | 0 | 1 | 0 | 0 |
| Chongsuvivatwong et al. (1996) [41] | I | 0 | 0 | 0 | 0 | 0 | 0 | 0 | 0 |
| Corrales et al. (2006) [61] | I | 0 | 0 | 0 | 0 | 0 | 0 | 0 | 0 |
| Cort et al. (1929) [20] | E | 0 | 0 | 0 | 0 | 0 | 1 | 0 | 0 |
| Curtale et al. (1999) [S24] | E | 0 | 1 | 0 | 0 | 0 | 0 | 1 | 0 |
| Curtale et al. (1998) [S25] | E | 0 | 0 | 0 | 1 | 0 | 0 | 1 | 0 |
| de Souza et al. (2007) [95] | I | 0 | 0 | 0 | 0 | 0 | 0 | 0 | 0 |
| Dolo et al. (1996) [S26] | E | 0 | 0 | 0 | 0 | 0 | 1 | 0 | 0 |
| Dumba et al. (2008) [S27] | E | 0 | 1 | 0 | 0 | 0 | 0 | 1 | 0 |
| Ekpo et al. (2008) [S28] | E | 0 | 0 | 1 | 0 | 0 | 0 | 1 | 0 |
| Ellis et al. (2007) [S29] | E | 0 | 0 | 0 | 0 | 0 | 1 | 0 | 0 |
| Embil et al. (1984) [S30] | E | 0 | 0 | 0 | 0 | 0 | 1 | 0 | 0 |
| Ensink et al. (2005) [34] | I | 0 | 0 | 0 | 0 | 0 | 0 | 0 | 0 |
| Erlanger et al. (2008) [46] | I | 0 | 0 | 0 | 0 | 0 | 0 | 0 | 0 |
| Escobedo et al. (2008) [S31] | E | 0 | 0 | 0 | 0 | 0 | 1 | 0 | 0 |
| Esrey et al. (1991) [27] | E | 1 | 0 | 0 | 0 | 0 | 0 | 0 | 0 |
| Eve et al. (1998) [S32] | E | 0 | 0 | 0 | 1 | 0 | 0 | 1 | 0 |
| Eyles et al. (1953) [S33] | E | 0 | 0 | 1 | 0 | 0 | 0 | 1 | 0 |
| Ezeagwuna et al. (2010) [S34] | E | 0 | 0 | 0 | 0 | 0 | 1 | 0 | 0 |
| Faust and Gonzalesmugaburu (1965) [S35] | E | 0 | 1 | 0 | 0 | 1 | 1 | 1 | 0 |
| Feachem et al. (1983) [S36] | E | 0 | 0 | 1 | 0 | 0 | 0 | 1 | 0 |
| Gamboa et al. (2003) [S37] | E | 0 | 0 | 0 | 0 | 0 | 1 | 0 | 0 |
| Gamboa et al. (1996) [S38] | E | 0 | 0 | 0 | 0 | 0 | 1 | 0 | 0 |
| Gbakima and Sahr (1995) [S39] | E | 0 | 0 | 0 | 0 | 0 | 1 | 0 | 0 |
| Gloor et al. (1970) [68] | I | 0 | 0 | 0 | 0 | 0 | 0 | 0 | 0 |
| Gross et al. (1989) [S40] | E | 0 | 1 | 0 | 0 | 0 | 1 | 1 | 0 |
| Gunawardena et al. (2004) [35] | I | 0 | 0 | 0 | 0 | 0 | 0 | 0 | 0 |
| Gunawardena et al. (2005) [36] | I | 0 | 0 | 0 | 0 | 0 | 0 | 0 | 0 |
| Hadidjaja et al. (1998) [S41] | E | 0 | 0 | 0 | 0 | 0 | 1 | 0 | 0 |
| Hagel et al. (1993) [67] | I | 0 | 0 | 0 | 0 | 0 | 0 | 0 | 0 |
| Hall et al. (1994) [S42] | E | 0 | 1 | 0 | 0 | 0 | 0 | 1 | 0 |
| Henry (1981) [S43] | E | 0 | 0 | 0 | 0 | 1 | 0 | 1 | 0 |
| Henry (1988) [S44] | E | 0 | 0 | 0 | 0 | 1 | 0 | 1 | 0 |
| Henry et al. (1993) [S45] | E | 0 | 0 | 0 | 0 | 1 | 0 | 1 | 0 |
| Holland et al. (1988) [60] | I | 0 | 0 | 0 | 0 | 0 | 0 | 0 | 0 |
| Holz (1963) [S46] | E | 0 | 0 | 0 | 0 | 0 | 1 | 0 | 0 |
| Hosain et al. (2003) [S47] | E | 0 | 1 | 0 | 0 | 0 | 0 | 1 | 0 |
| House (1982) [S48] | E | 1 | 0 | 0 | 0 | 0 | 0 | 0 | 0 |
| Hughes et al. (2004) [80] | E | 0 | 0 | 0 | 0 | 0 | 0 | 0 | 1 (school latrines) |
| Huttly (1990) [S49] | E | 1 | 0 | 0 | 0 | 0 | 0 | 0 | 0 |
| Idowu and Rowland (2006) [S50] | E | 0 | 0 | 0 | 0 | 0 | 1 | 0 | 0 |
| Ilechukwu et al. (2010) [59] | I | 0 | 0 | 0 | 0 | 0 | 0 | 0 | 0 |
| Ittiravivongs et al. (1992) [S51] | E | 0 | 0 | 1 | 0 | 0 | 0 | 1 | 0 |
| Iushko (2006) [S52] | E | 0 | 0 | 0 | 0 | 0 | 1 | 0 | 0 |
| Jombo et al. (2007) [54] | I | 0 | 0 | 0 | 0 | 0 | 0 | 0 | 0 |
| Jombo et al. (2010) [S53] | E | 0 | 1 | 0 | 0 | 0 | 0 | 1 | 0 |
| Khalil et al. (1931) [21] | E | 0 | 0 | 0 | 0 | 0 | 1 | 0 | 0 |
| Khan (1987) [S54] | E | 0 | 0 | 0 | 1 | 0 | 0 | 1 | 0 |
| Khromenkova et al. (2008) [S55] | E | 0 | 0 | 0 | 0 | 0 | 1 | 0 | 0 |
| Kightlinger et al. (1998) [50] | I | 0 | 0 | 0 | 0 | 0 | 0 | 0 | 0 |
| Knopp et al. (2010) [58] | I | 0 | 0 | 0 | 0 | 0 | 0 | 0 | 0 |
| Koroma et al. (2010) [S56] | E | 0 | 0 | 0 | 0 | 0 | 1 | 0 | 0 |
| Le Hung et al. (2005) [S57] | E | 0 | 0 | 0 | 0 | 1 | 0 | 1 | 0 |
| Legesse et al. (2010) [S58] | E | 0 | 0 | 0 | 0 | 0 | 1 | 0 | 0 |
| Longuet et al. (1993) [S59] | E | 0 | 0 | 0 | 0 | 0 | 1 | 0 | 0 |
| Ludwig et al. (1999) [S60] | E | 0 | 1 | 0 | 0 | 0 | 1 | 1 | 0 |
| Luong (2003) [S61] | E | 1 | 0 | 0 | 0 | 0 | 0 | 0 | 0 |
| Mangali et al. (1993) [S62] | E | 0 | 0 | 0 | 1 | 0 | 0 | 1 | 0 |
| Mangali et al. (1994) [S63] | E | 0 | 0 | 1 | 0 | 0 | 0 | 1 | 0 |
| Martin (1972) [S64] | E | 0 | 0 | 0 | 0 | 0 | 1 | 0 | 0 |
| Mascarini-Serra et al. (2010) [S65] | E | 0 | 0 | 0 | 0 | 1 | 0 | 1 | 0 |
| Massara and Enk (2004) [S66] | E | 1 | 0 | 0 | 0 | 0 | 0 | 0 | 0 |
| Matthys et al. (2007) [55] | I | 0 | 0 | 0 | 0 | 0 | 0 | 0 | 0 |
| Menghi et al. (2007) [S67] | E | 0 | 0 | 0 | 0 | 0 | 1 | 0 | 0 |
| Messou et al. (1997) [S68] | E | 0 | 0 | 0 | 0 | 1 | 0 | 1 | 0 |
| Moore et al. (1965) [S69] | E | 0 | 0 | 0 | 0 | 1 | 0 | 1 | 0 |
| Moraes and Cairncross (2004) [S70] | E | 0 | 0 | 0 | 0 | 1 | 1 | 1 | 0 |
| Moraes et al. (2004) [S71] | E | 0 | 0 | 0 | 0 | 1 | 0 | 1 | 0 |
| Moraes Neto et al. (2010) [S72] | E | 0 | 0 | 0 | 0 | 0 | 1 | 0 | 0 |
| Morales-Espinoza et al. (2003) [63] | I | 0 | 0 | 0 | 0 | 0 | 0 | 0 | 0 |
| Mordi and Okaka (2009) [S73] | E | 0 | 0 | 0 | 0 | 0 | 1 | 0 | 0 |
| Muennoo et al. (1997) [S74] | E | 0 | 0 | 0 | 0 | 1 | 0 | 1 | 0 |
| Muller (1988) [S75] | E | 0 | 0 | 1 | 0 | 0 | 0 | 1 | 0 |
| Muller et al. (1989) [S76] | E | 0 | 0 | 1 | 0 | 0 | 0 | 1 | 0 |
| Naish et al. (2004) [S77] | E | 0 | 0 | 0 | 0 | 0 | 1 | 0 | 0 |
| Navarrete and Torres (1994) [S78] | E | 0 | 0 | 0 | 0 | 0 | 1 | 0 | 0 |
| Nguyen et al. (2006) [47] | I | 0 | 0 | 0 | 0 | 0 | 0 | 0 | 0 |
| Nishiura et al. (2002) [37] | I | 0 | 0 | 0 | 0 | 0 | 0 | 0 | 0 |
| Nitin et al. (2007) [S79] | E | 0 | 1 | 0 | 0 | 0 | 0 | 1 | 0 |
| Olsen et al. (2001) [51] | I | 0 | 0 | 0 | 0 | 0 | 0 | 0 | 0 |
| Onwuliri et al. (1992) [S80] | E | 0 | 1 | 0 | 0 | 0 | 0 | 1 | 0 |
| Oyewole et al. (2002) [S81] | E | 0 | 0 | 0 | 0 | 1 | 1 | 1 | 0 |
| Pastushchak (1963) [S82] | E | 0 | 0 | 0 | 0 | 0 | 1 | 0 | 0 |
| Pawlowski (1985) [S83] | E | 1 | 0 | 0 | 0 | 0 | 0 | 0 | 0 |
| Pawlowski (1983) [S84] | E | 1 | 0 | 0 | 0 | 0 | 0 | 0 | 0 |
| Pezzani et al. (1996) [S85] | E | 0 | 0 | 0 | 0 | 0 | 1 | 0 | 0 |
| Pimentel et al. (1961) [S86] | E | 0 | 0 | 0 | 0 | 0 | 1 | 0 | 0 |
| Poggensee et al. (2005) [S87] | E | 0 | 0 | 0 | 0 | 1 | 0 | 1 | 0 |
| Prüss et al. (2002) [S88] | E | 1 | 0 | 0 | 0 | 0 | 0 | 0 | 0 |
| Rai et al. (1997) [S89] | E | 0 | 0 | 0 | 0 | 0 | 0 | 0 | 1 (unable to obtain this reference) |
| Rai et al. (2005) [S90] | E | 0 | 0 | 0 | 0 | 0 | 0 | 0 | 1 (unable to obtain this reference) |
| Raja'a et al. (2001) [43] | I | 0 | 0 | 0 | 0 | 0 | 0 | 0 | 0 |
| Rajeswari et al. (1994) [S91] | E | 0 | 1 | 0 | 0 | 0 | 0 | 1 | 0 |
| Rinne et al. (2005) [S92] | E | 0 | 0 | 0 | 0 | 0 | 1 | 0 | 0 |
| Roller and Vuturo (1971) [S93] | E | 0 | 0 | 0 | 0 | 0 | 1 | 0 | 0 |
| Romanenko (1980) [S94] | E | 0 | 0 | 0 | 0 | 0 | 1 | 0 | 0 |
| Rosewell et al. (2010) [S95] | E | 0 | 0 | 0 | 0 | 0 | 1 | 0 | 0 |
| Sadun et al. (1954) [S96] | E | 0 | 0 | 0 | 0 | 1 | 0 | 1 | 0 |
| Sahba and Arfaa (1967) [97] | E | 0 | 0 | 0 | 0 | 1 | 0 | 1 | 0 |
| Schliessmann et al. (1958) [72] | E | 0 | 0 | 1 | 0 | 0 | 0 | 1 | 0 |
| Scolari et al. (2000) [S98] | E | 0 | 0 | 0 | 0 | 0 | 1 | 0 | 0 |
| Scott and Barlow (1938) [70] | E | 0 | 0 | 0 | 0 | 1 | 0 | 1 | 0 |
| Shang et al. (2010) [S99] | E | 0 | 0 | 0 | 0 | 0 | 1 | 0 | 0 |
| Singh et al. (2010) [S100] | E | 0 | 0 | 1 | 0 | 0 | 0 | 1 | 0 |
| Sorensen et al. (1994) [S101] | E | 0 | 0 | 0 | 1 | 0 | 0 | 1 | 0 |
| Steinmann et al. (2006) [S102] | E | 1 | 1 | 0 | 0 | 0 | 1 | 1 | 0 |
| Steinmann et al. (2010) [48] | I | 0 | 0 | 0 | 0 | 0 | 0 | 0 | 0 |
| Stephenson et al. (1983) [49] | I | 0 | 0 | 0 | 0 | 0 | 0 | 0 | 0 |
| Stothard et al. (2008) [56] | I | 0 | 0 | 0 | 0 | 0 | 0 | 0 | 0 |
| Stürchler et al. (1980) [83] | E | 0 | 0 | 0 | 0 | 0 | 1 | 0 | 0 |
| Sun et al. (2003) [38] | I | 0 | 0 | 0 | 0 | 0 | 0 | 0 | 0 |
| Sweet et al. (1929) [S103] | E | 0 | 0 | 0 | 0 | 0 | 1 | 0 | 0 |
| Toma et al. (1999) [39] | I | 0 | 0 | 0 | 0 | 0 | 0 | 0 | 0 |
| Torres et al. (1997) [66] | I | 0 | 0 | 0 | 0 | 0 | 0 | 0 | 0 |
| Trang et al. (2006) [45] | I | 0 | 0 | 0 | 0 | 0 | 0 | 0 | 0 |
| Trang et al. (2007) [44] | I | 0 | 0 | 0 | 0 | 0 | 0 | 0 | 0 |
| Traub et al. (2004) [40] | I | 0 | 0 | 0 | 0 | 0 | 0 | 0 | 0 |
| Tshikuka et al. (1995) [S104] | E | 0 | 0 | 0 | 1 | 0 | 0 | 1 | 0 |
| Udonsi and Ogan (1993) [S105] | E | 0 | 0 | 0 | 0 | 1 | 0 | 1 | 0 |
| Ugbomoiko et al. (2009) [57] | I | 0 | 0 | 0 | 0 | 0 | 0 | 0 | 0 |
| Ulukanligil and Seyrek (2003) [S106] | E | 0 | 0 | 1 | 0 | 0 | 1 | 1 | 0 |
| Verle et al. (2003) [S107] | E | 0 | 0 | 0 | 0 | 0 | 1 | 0 | 0 |
| Wagbatsoma and Aimiuwu (2008) [S108] | E | 0 | 0 | 0 | 0 | 0 | 1 | 0 | 0 |
| Waisley (2000) [S109] | E | 1 | 0 | 0 | 0 | 0 | 0 | 0 | 0 |
| Wang et al. (2009) [23] | E | 1 | 0 | 0 | 0 | 1 | 0 | 1 | 0 |
| Wani et al. (2007) [S110] | E | 0 | 0 | 1 | 0 | 0 | 0 | 1 | 0 |
| Wördemann et al. (2006) [62] | I | 0 | 0 | 0 | 0 | 0 | 0 | 0 | 0 |
| Yajima et al. (2009) [42] | I | 0 | 0 | 0 | 0 | 0 | 0 | 0 | 0 |
| Yassin et al. (1999) [S111] | E | 0 | 0 | 0 | 0 | 0 | 1 | 0 | 0 |
| Yokogawa (1976) [S112] | E | 1 | 0 | 0 | 0 | 0 | 0 | 0 | 0 |
| Zheng et al. (2009) [S113] | E | 1 | 0 | 0 | 0 | 0 | 0 | 0 | 0 |
|  |  |  |  |  |  |  |  |  |  |
| Total | 36 | 21 | 14 | 13 | 6 | 21 | 59 | 53 | 3 |

**References**

S1. Abu Mourad TA (2004) Palestinian refugee conditions associated with intestinal parasites and diarrhoea: Nuseirat refugee camp as a case study. Public Health 118: 131-142.

S2. Acka CA, Raso G, N'Goran EK, Tschannen AB, Bogoch II, et al. (2010) Parasitic worms: knowledge, attitudes, and practices in western Côte d’Ivoire with implications for integrated control. PLoS Negl Trop Dis 4: e910.

S3. Adeoye GO, Osayemi CO, Oteniya O, Onyemekeihia SO (2007) Epidemiological studies of intestinal helminthes and malaria among children in Lagos, Nigeria. Pak J Biol Sci 10: 2208-2212.

S4. Aimpun P, Hshieh P (2004) Survey for intestinal parasites in Belize, Central America. Southeast Asian J Trop Med Public Health 35: 506-511.

S5. Albonico M, Montresor A, Crompton DW, Savioli L (2006) Intervention for the control of soil-transmitted helminthiasis in the community. Adv Parasitol 61: 311-348.

S6. Amuta EU, Houmsou RS, Mker SD (2010) Knowledge and risk factors of intestinal parasitic infections among women in Makurdi, Benue State. Asian Pac J Trop Med 3 : 993-996.

S7. Anantaphruti MT, Nuamtanong S, Muennoo C, Sanguankiat S, Pubampen S (2000) *Strongyloides stercoralis* infection and chronological changes of other soil-transmitted helminthiases in an endemic area of southern Thailand. Southeast Asian J Trop Med Public Health 31: 378-382.

S8. Anthony G, Deming M, Dorkenoo AM, Morgah K, Verani J, et al. (2009) The integration of neglected diseases: three years of experience in Togo. Am J Trop Med Hyg 81: 327-328.

S9. Antunes M, Gomonda E, Seni A, Baldessin F, Beltramello C, et al. (2009) High prevalence of soil-transmitted helminths, low prevalence of *Schistosoma haematobium* and lack of *Schistosoma mansoni* infections in a suburban population of Beira, Mozambique. Trop Med Int Health 14: 197.

S10. Barreto M, Genser B, Strina A, Teixeira M, Assis A, et al. (2010) Impact of a citywide sanitation program in Northeast Brazil on intestinal parasites infection in young children. Environ Health Perspect 118: 1637-1642.

S11. Bartlett S (2003) Water, sanitation and urban children: the need to go beyond “improved” provision. Environ Urban 15: 57-70.

S12. Bath JL, Eneh PN, Bakken AJ, Knox ME, Schiedt MD, et al. (2010) The impact of perception and knowledge on the treatment and prevention of intestinal worms in the Manikganj district of Bangladesh. Yale J Biol Med 83: 171-184.

S13. Bdir S, Adwan G (2010) Prevalence of intestinal parasitic infections in Jenin governorate, Palestine: a 10-year retrospective study. Asian Pac J Trop Med 3: 745-747.

S14. Belyhun Y, Medhin G, Amberbir A, Erko B, Hanlon C, et al. (2010) Prevalence and risk factors for soil-transmitted helminth infection in mothers and their infants in Butajira, Ethiopia: a population based study. BMC Public Health 10: 21.

S15. Blum D, Feachem RG (1983) Measuring the impact of water supply and sanitation investments on diarrhoeal diseases: problems of methodology. Int J Epidemiol 12: 357-365.

S16. Boia MN, da Motta LP, Salazar MD, Mutis MP, Coutinho RB, et al. (1999) Cross-sectional study of intestinal parasites and Chagas’ disease in the municipality of Novo Airao, State of Amazonas, Brazil. Cad Saude Publica 15: 497-504 (in Portuguese).

S17. Borda CE, Rea MJ, Rosa JR, Maidana C (1996) Intestinal parasitism in San Cayetano, Corrientes, Argentina. Bull Pan Am Health Organ 30: 227-233.

S18. Bradley M, Chandiwana SK, Bundy DA (1993) The epidemiology and control of hookworm infection in the Burma valley area of Zimbabwe. Trans R Soc Trop Med Hyg 87: 145-147.

S19. Cairncross S (1989) Water supply and sanitation: an agenda for research. J Trop Med Hyg 92: 301-314.

S20. Cairncross S (2003) Sanitation in the developing world: current status and future solutions. Int J Environ Health Res 13 (Suppl 1): S123-131.

S21. Silva RCR, Barreto ML, Assis AMO, Santana MLP, Parraga IM, et al. (2007) The relative influence of polyparasitism, environment, and host factors on schistosome infection. Am J Trop Med Hyg 77: 672-675.

S22. Chandiwana SK, Bradley M, Chombo F (1989) Hookworm and roundworm infections in farm-worker communities in the large-scale agricultural sector in Zimbabwe. J Trop Med Hyg 92: 338-344.

S23. Chandler AC (1954) A comparison of helminthic and protozoan infections in two Egyptian villages two years after the installation of sanitary improvements in one of them. Am J Trop Med Hyg 3: 59-73.

S24. Curtale F, Pezzotti P, Saad YS, Aloi A (1999) An analysis of individual, household, and environmental risk factors for intestinal helminth infection among children in Qena governorate, Upper Egypt. J Trop Pediatr 45: 14-17.

S25. Curtale F, Shamy MY, Zaki A, Abdel-Fattah M, Rocchi G (1998) Different patterns of intestinal helminth infection among young workers in urban and rural areas of Alexandria governorate, Egypt. Parassitologia 40: 251-254.

S26. Dolo A, Cancrini G, Traore F, Traore S, Kassambara L, et al. (1996) Protozoan infections and intestinal helminthiasis among the population of a village in the northern Sudan savannah area of Mali (West Africa). Parassitologia 38: 585-589 (in French).

S27. Dumba R, Kaddu JB, Mangen FW (2008) Intestinal helminths in Luweero district, Uganda. Afr Health Sci 8: 90-96.

S28. Ekpo U, Odoemene S, Mafiana C, Sam-Wobo S (2008) Helminthiasis and hygiene conditions of schools in Ikenne, Ogun state, Nigeria. PLoS Negl Trop Dis 2: e146.

S29. Ellis MK, Raso G, Li Y, Rong Z, Chen H, et al. (2007) Familial aggregation of human susceptibility to co- and multiple helminth infections in a population from the Poyang Lake region, China. Int J Parasitol 37: 1153-1161.

S30. Embil JA, Pereira LH, White FM, Garner JB, Manuel FR (1984) Prevalence of *Ascaris lumbricoides* infection in a small Nova Scotian community. Am J Trop Med Hyg 33: 595-598.

S31. Escobedo AA, Canete R, Nunez FA (2008) Prevalence, risk factors and clinical features associated with intestinal parasitic infections in children from San Juan y Martinez, Pinar del Rio, Cuba. West Indian Med J 57: 377-382.

S32. Eve E, Ferraz E, Thatcher VE (1998) Parasitic infections in villagers from three districts of the Brazilian Amazon. Ann Trop Med Parasitol 92: 79-87.

S33. Eyles DE, Jones FE, Smith CS (1953) A study of *Endamoeba histolytica* and other intestinal parasites in a rural West Tennessee community. Am J Trop Med Hyg 2: 173-190.

S34. Ezeagwuna DA, Okwelogu IS, Ekejindu IM, Ogbuagu CN (2010) The prevalence and socio-economic factors of intestinal helminth infections among primary school pupils in Ozubulu, Anambra state, Nigeria. Int J Epidemiol Volume 9 Number 1

S35. Faust EC, Gonzales-Mugaburu L (1965) Parasitologic surveys in Cali, Dempartmento del Valle, Colombia. XI. Intestinal parasites in Ward Silo’e, Cali, during a four-year period 1956-1960. Am J Trop Med Hyg 14: 276-289.

S36. Feachem RG, Guy MW, Harrison S, Iwugo KO, Marshall T, et al. (1983) Excreta disposal facilities and intestinal parasitism in urban Africa: preliminary studies in Botswana, Ghana and Zambia. Trans R Soc Trop Med Hyg 77: 515-521.

S37. Gamboa MI, Basualdo JA, Cordoba MA, Pezzani BC, Minvielle MC, et al. (2003) Distribution of intestinal parasitoses in relation to environmental and sociocultural parameters in La Plata, Argentina. J Helminthol 77: 15-20.

S38. Gamboa MI, Basualdo JA, Kozubsky L, Costas ME, Cueto ER, et al. (1996) Intestinal parasites in two periurban populations in La Plata, Argentina. Bol Chil Parasitol 51: 37-41 (in Spanish).

S39. Gbakima AA, Sahr F (1995) Intestinal parasitic infections among rural farming communities in eastern Sierra Leone. Afr J Med Med Sci 24: 195-200.

S40. Gross R, Schell B, Molina MC, Leao MA, Strack U (1989) The impact of improvement of water supply and sanitation facilities on diarrhea and intestinal parasites: a Brazilian experience with children in two low-income urban communities. Rev Saude Publica 23: 214-220.

S41. Hadidjaja P, Bonang E, Suyardi MA, Abidin SA, Ismid IS, et al. (1998) The effect of intervention methods on nutritional status and cognitive function of primary school children infected with *Ascaris lumbricoides*. Am J Trop Med Hyg 59: 791-795.

S42. Hall A, Conway DJ, Anwar KS, Rahman ML (1994) *Strongyloides stercoralis* in an urban slum community in Bangladesh: factors independently associated with infection. Trans R Soc Trop Med Hyg 88: 527-530.

S43. Henry FJ (1981) Environmental sanitation infection and nutritional status of infants in rural St. Lucia, West Indies. Trans R Soc Trop Med Hyg 75: 507-513.

S44. Henry FJ (1988) Reinfection with *Ascaris lumbricoides* after chemotherapy: a comparative study in three villages with varying sanitation. Trans R Soc Trop Med Hyg 82: 460-464.

S45. Henry FJ, Huttly SR, Ahmed MU, Alam A (1993) Effect of chemotherapy on helminth reinfection in slums and villages in Bangladesh. Southeast Asian J Trop Med Public Health 24: 307-312.

S46. Holz (1963) A pilot project for the control of ancylostomiasis in rural areas of Indonesia. Z Tropenmed Parasitol 14: 519-525.

S47. Hosain G, Saha S, Begum A (2003) Impact of sanitation and health education on intestinal parasite infection among primary school aged children of Sherpur, Bangladesh. Trop Doct 33: 139-143.

S48. House RV (1982) A brief history of hookworm disease in North Carolina. N C Med J 43: 765-768.

S49. Huttly SR (1990) The impact of inadequate sanitary conditions on health in developing countries. World Health Stat Q 43: 118-126.

S50. Idowu OA, Rowland SA (2006) Oral fecal parasites and personal hygiene of food handlers in Abeokuta, Nigeria. Afr Health Sci 6: 160-164.

S51. Ittiravivongs A, Kasornkul C, Soyraya R, Soyraya J, Pattara-arechachai J (1992) Assessment of sanitation conditions by qualitative sanitation measurement. Southeast Asian J Trop Med Public Health 23: 212-218.

S52. Iushko LA (2006) Sanitary-helminthological status of the environment in Yalta. Med Parazitol (Mosk): 39-40 (in Russian).

S53. Jombo GT, Damen JG, Safiyanu H, Odey F, Mbaawuaga EM (2010) Human intestinal parasitism, potable water availability and methods of sewage disposal among nomadic Fulanis in Kuraje rural settlement of Zamfara state. Asian Pac J Trop Med 3: 491-493.

S54. Khan MU (1987) Limitation of communal latrines in changing the prevalence of parasites and diarrhoeal attack rate in Dhaka peri-urban slums. Environ Pollut 47: 187-194.

S55. Khromenkova EP, Vaserin I, Romanenko NA, Dimidova LL, Upyrev AV, et al. (2008) The sanitary and parasitological characteristics of environmental objects in the south of Russia. Wastewaters and their precipitations. Med Parazitol (Mosk): 25-29 (in Russian).

S56. Koroma JB, Peterson J, Gbakima AA, Nylander FE, Sahr F, et al. (2010) Geographical distribution of intestinal schistosomiasis and soil-transmitted helminthiasis and preventive chemotherapy strategies in Sierra Leone. PLoS Negl Trop Dis 4: e891.

S57. Hung LQ, de Vries PJ, Giao PT, Binh TQ, Nam NV, et al. (2005) Intestinal helminth infection in an ethnic minority commune in southern Vietnam. Southeast Asian J Trop Med Public Health 36: 623-628.

S58. Legesse L, Erko B, Hailu A (2010) Current status of intestinal schistosomiasis and soil-transmitted helminthiasis among primary school children in Adwa town, northern Ethiopia. Ethiop J Health Dev 24: 191-197.

S59. Longuet C, David C, Klotz F (1993) Intestinal parasitoses in the south-eastern district of the Dominican Republic. Med Trop (Mars) 53: 337-340 (in French).

S60. Ludwig KM, Frei F, Alvares F, Ribeiro-Paes JT (1999) Correlation between sanitation conditions and intestinal parasitosis in the population of Assis, State of Sao Paulo. Rev Soc Bras Med Trop 32: 547-555 (in Portugese).

S61. Luong TV (2003) De-worming school children and hygiene intervention. Int J Environ Health Res 13 (Suppl 1): S153-159.

S62. Mangali A, Sasabone P, Abadi K, Hasegawa H, Toma T, et al. (1993) Intestinal parasitic infections in Campalagian district, south Sulawesi, Indonesia. Southeast Asian J Trop Med Public Health 24: 313-320.

S63. Mangali A, Sasabone P, Abadi K, Hasegawa H, Toma T, et al. (1994) Prevalence of intestinal helminthic infections in Kao district, north Halmahera, Indonesia. Southeast Asian J Trop Med Public Health 25: 737-744.

S64. Martin LK (1972) Hookworm in Georgia. II. Survey of intestinal helminth infections in members of rural households of southeastern Georgia. Am J Trop Med Hyg 21: 930-943.

S65. Mascarini-Serra L, Telles C, Prado M, Mattos S, Strina A, et al. (2010) Reductions in the prevalence and incidence of geohelminth infections following a city-wide sanitation program in a Brazilian urban centre. PLoS Negl Trop Dis 4: e588.

S66. Massara C, Enk M (2004) Treatment options in the management of *Ascaris lumbricoides*. Expert Opin Pharmacother 5: 529-539.

S67. Menghi CI, Iuvaro FR, Dellacasa MA, Gattai CL (2007) Survey of intestinal parasites among an aboriginal community in Salta. Medicina-Buenos Aires 67: 705-708.

S68. Messou E, Sangare SV, Josseran R, Le Corre C, Guelain J (1997) Impact of improved sanitary conditions and domestic hygiene on the incidence of ascaridiasis and ancylostomiasis in children two to four years old in the rural zones of Ivory Coast. Bull Soc Pathol Exot Filiales 90: 48-50 (in French).

S69. Moore HA, De la Cruz E, Vargas-Mendez O (1965) Diarrheal disease studies in Costa Rica. IV. The influence of sanitation upon the prevalence of intestinal infection and diarrheal disease. Am J Epidemiol 82: 162-184.

S70. Moraes LR, Cairncross S (2004) Environmental interventions and the pattern of geohelminth infections in Salvador, Brazil. Parasitology 129: 223-232.

S71. Moraes LR, Cancio J, Cairncross S (2004) Impact of drainage and sewerage on intestinal nematode infections in poor urban areas in Salvador, Brazil. Trans R Soc Trop Med Hyg 98: 197-204.

S72. Moraes Neto AH, Pereira AP, Alencar MF, Souza PR Jr, Dias RC, et al. (2010) Prevalence of intestinal parasites versus knowledge, attitudes, and practices of inhabitants of low-income communities of Campos dos Goytacazes, Rio de Janeiro State, Brazil. Parasitol Res 107: 295-307.

S73. Mordi RM, Okaka CE (2009) Prevalence of intestinal parasites in Edo state. Int J Health Res 2: 253-257.

S74. Muennoo C, Achwanichkul W, Sa-nguankiat S, Pubampen S, Maipanich W, et al. (1997) The impact of primary health care intervention on reinfection of soil-transmitted helminths in the community. Southeast Asian J Trop Med Public Health 28: 816-819.

S75. Muller M (1988) Increasing the effectiveness of a latrine programme. World Health Forum 9: 345-351.

S76. Muller M, Sanchez RM, Suswillo RR (1989) Evaluation of a sanitation programme using eggs of *Ascaris lumbricoides* in household yard soils as indicators. J Trop Med Hyg 92: 10-16.

S77. Naish S, McCarthy J, Williams GM (2004) Prevalence, intensity and risk factors for soil-transmitted helminth infection in a South Indian fishing village. Acta Trop 91: 177-187.

S78. Navarrete N, Torres P (1994) Prevalence of infection by intestinal helminths and protozoa in school children from a coastal locality in the province of Valdivia, Chile. Bol Chil Parasitol 49: 79-80 (in Spanish).

S79. Nitin S, Venkatesh V, Husain N, Masood J, Agarwal GG (2007) Overview of intestinal parasitic prevalence in rural and urban population in Lucknow, north India. J Commun Dis 39: 217-223.

S80. Onwuliri CO, Imandeh NG, Okwuosa VN (1992) Human helminthosis in a rural community of Plateau state, Nigeria. Angew Parasitol 33: 211-216.

S81. Oyewole F, Ariyo F, Sanyaolu A, Oyibo WA, Faweya T, et al. (2002) Intestinal helminthiases and their control with albendazole among primary schoolchildren in riverine communities of Ondo State, Nigeria. Southeast Asian J Trop Med Public Health 33: 214-217.

S82. Pastushchak GI (1963) On methods of studying the effectiveness of refuse disposal in populated areas. Gig Sanit 28: 76-81.

S83. Pawlowski ZS (1983) Intestinal parasitic infections as a public health problem. Parassitologia 25: 141-150.

S84. Pawlowski ZS (1985) Ascariasis control. World Health Forum 6: 254-256.

S85. Pezzani BC, Minvielle MC, De L, Radman N, Iacoy P, et al. (1996) Intestinal parasite infections in a periurban community from the province of Buenos Aires, Argentina. Bol Chil Parasitol 51: 42-45 (in Spanish).

S86. Pimentel D, Gerhardt CE, Williams ER, White P, Ferguson FF (1961) Aspects of schistosomal endemicity in three Puerto Rican watersheds. Am J Trop Med Hyg 10: 523-529.

S87. Poggensee G, Krantz I, Nordin P, Mtweve S, Ahlberg B, et al. (2005) A six-year follow-up of schoolchildren for urinary and intestinal schistosomiasis and soil-transmitted helminthiasis in northern Tanzania. Acta Trop 93: 131-140.

S88. Prüss A, Kay D, Fewtrell L, Bartram J (2002) Estimating the burden of disease from water, sanitation, and hygiene at a global level. Environ Health Perspect 110: 537-542.

S89. Rai SK, Hirai K, Ohno Y, Matsumura T (1997) Village health and sanitary profile from eastern hilly region, Nepal. Kobe J Med Sci 43: 121-133.

S90. Rai D, Rai S, Sharma B, Ghimire P, Bhatta D (2005) Factors associated with intestinal parasitic infection among school children in a rural area of Kathmandu valley, Nepal. Nepal Med Coll J 7: 43-46.

S91. Rajeswari B, Sinniah B, Hussein H (1994) Socio-economic factors associated with intestinal parasites among children living in Gombak, Malaysia. Asia Pac J Public Health 7: 21-25.

S92. Rinne S, Rodas EJ, Galer-Unti R, Glickman N, Glickman LT (2005) Prevalence and risk factors for protozoan and nematode infections among children in an Ecuadorian highland community. Trans R Soc Trop Med Hyg 99: 585-592.

S93. Roller OE, Vuturo AF (1971) Rural latrine systems, a pilot project in the Ryukyu Islands. HSMHA Health Rep 86: 637-640.

S94. Romanenko NA (1980) Current status and prospects for the development of sanitary helminthology in the USSR. Med Parazitol (Mosk) 49: 24-28 (in Russian).

S95. Rosewell A, Robleto G, Rodriguez G, Barragne-Bigot P, Amador JJ, et al. (2010) Soil-transmitted helminth infection and urbanization in 880 primary school children in Nicaragua, 2005. Trop Doct 40: 141-143.

S96. Sadun EH, Vajrasthira S, Maiphoom C (1954) The effect of treatment and sanitation on hookworm infection in Cholburi province (Central Thailand). Am J Trop Med Hyg 3: 764-772.

S97. Sahba GH, Arfaa F (1967) The effect of sanitation on ascariasis in an Iranian village. J Trop Med Hyg 70: 37-39.

S98. Scolari C, Torti C, Beltrame A, Matteelli A, Castelli F, et al. (2000) Prevalence and distribution of soil-transmitted helminth (STH) infections in urban and indigenous schoolchildren in Ortigueria, state of Parana, Brasil: implications for control. Trop Med Int Health 5: 302-307.

S99. Shang Y, Tang LH, Zhou SS, Chen YD, Yang YC, et al. (2010) Stunting and soil-transmitted-helminth infections among school-age pupils in rural areas of southern China. Parasit Vectors 3: 97.

S100. Singh C, Zargar SA, Masoodi I, Shoukat A, Ahmad B (2010) Predictors of intestinal parasitosis in school children of Kashmir: a prospective study. Trop Gastroenterol 31: 105-107.

S101. Sorensen E, Ismail M, Amarasinghe DKC, Hettiarachchi I, Dassenaieke CTS (1994) The effect of the availability of latrines on soil-transmitted nematode infections in the plantation sector in Sri Lanka. Am J Trop Med Hyg 51: 36-39.

S102. Steinmann P, Keiser J, Bos R, Tanner M, Utzinger J (2006) Schistosomiasis and water resources development: systematic review, meta-analysis, and estimates of people at risk. Lancet Infect Dis 6: 411-425.

S103. Sweet WC, Cort WW, Schapiro L, Stoll NR, Riley WA (1929) A study of the effect of treatment and sanitation on the level of hookworm infestation in certain areas in Panama. Am J Hyg 9: 98-138.

S104. Tshikuka JG, Scott ME, Gray-Donald K (1995) *Ascaris lumbricoides* infection and environmental risk factors in an urban African setting. Ann Trop Med Parasitol 89: 505-514.

S105. Udonsi JK, Ogan VN (1993) Assessment of the effectiveness of primary health care interventions in the control of three intestinal nematode infections in rural communities. Public Health 107: 53-60.

S106. Ulukanligil M, Seyrek A (2003) Demographic and parasitic infection status of schoolchildren and sanitary conditions of schools in Sanliurfa, Turkey. BMC Public Health 3: 29.

S107. Verle P, Kongs A, De NV, Thieu NQ, Depraetere K, et al. (2003) Prevalence of intestinal parasitic infections in northern Vietnam. Trop Med Int Health 8: 961-964.

S108. Wagbatsoma VA, Aimiuwu U (2008) Sanitary provision and helminthiasis among school children in Benin City, Nigeria. Niger Postgrad Med J 15: 105-111.

S109. Waisley T (2000) Public health programs in early twentieth-century Louisiana. La Hist 41: 41-69.

S110. Wani S, Ahmad F, Zargar S, Ahmad Z, Ahmad P, et al. (2007) Prevalence of intestinal parasites and associated risk factors among schoolchildren in Srinagar City, Kashmir, India. J Parasitol 93: 1541-1543.

S111. Yassin MM, Shubair ME, al-Hindi AI, Jadallah SY (1999) Prevalence of intestinal parasites among school children in Gaza City, Gaza Strip. J Egypt Soc Parasitol 29: 365-373.

S112. Yokogawa M (1976) Programme of schistosomiasis control in Japan. Southeast Asian J Trop Med Public Health 7: 322-329.

S113. Zheng Q, Chen Y, Zhang HB, Chen JX, Zhou XN (2009) The control of hookworm infection in China. Parasit Vectors 2: 44.
