# Supplementary material for: Effect of Sanitation on Soil-Transmitted Helminth Infection: Systematic Review and Meta-Analysis
Source: PLoS Med. 2012 Jan 24;9(1):e1001162. doi: 10.1371/journal.pmed.1001162 (PMC3265535; doi:10.1371/journal.pmed.1001162)
Supplement: Text S2 — Study protocol for systematic review and meta-analysis to determine the effect of sanitation on soil-transmitted helminth infection. (DOC) [file pmed.1001162.s003.doc]

**Text S2** Study protocol for systematic review and meta-analysis to determine the effect of sanitation on soil-transmitted helminth infection

(Ziegelbauer, Speich et al. “Effect of Sanitation on Soil-Transmitted Helminth Infection: Systematic Review and Meta-Analysis; PMEDICINE-D-10-00012R2)

**Objective**

To examine the effect of availability and/or use of sanitation against the three common soil-transmitted helminth infections (i.e., *Ascaris lumbricoides*, *Trichuris trichiura*, and hookworm).

**Inclusion Criteria**

*Study type*

- All study types will be eligible to enter our meta-analysis.
- Based on two existing reviews (Esrey et al. 1991; Asaolu and Ofoezie 2003), we assume that there will be no or only very limited evidence obtained from randomized controlled trials (RCTs) assessing the effect of sanitation against soil-transmitted helminth infections. Moreover, we assume that an important drawback of RCTs and intervention studies is that results would be difficult to pool due to important differences in methodology, study design, interventions, and coverage.
- Hence, we will include all studies which report data on sanitation availability and/or use and soil-transmitted helminth infection on individual level.

*Participants*

- There will be no restriction on age or special high-risk groups (e.g., school-aged children).
- If possible, we will perform sub-group analyses (e.g., different age groups, geography, etc.).

*Parasites*

The review will focus on the most common soil-transmitted helminth species, namely:

- Roundworm (*Ascaris lumbricoides*).
- Whipeworm (*Trichuris trichiura*).
- Hookworm (two species: *Ancylostoma duodenale* and *Necator americanus*).

If possible, we will also include *Strongyloides stercoralis*.

*Sanitation*

- Studies presenting data on the availability and/or use of sanitation facilities will be included. The definition of sanitation will be broad, and hence, all types of latrines (e.g., pit latrines, ventilated improved pit (VIP) latrines, flush latrines, etc.) are eligible to enter our study. Open defecation will be defined as no sanitation.
- The same wide definition will apply for the use on individual level (e.g., assessed through a questionnaire; “do you use a sanitary facility?” or “where do you defecate?”). Therefore, if a study only mentions availability or use of sanitation without specifying its type, the study will also be included.
- In a next step different toilet types or coverage levels will be considered. Here, studies reporting data on the same toilet type (e.g., pit latrine, flush toilet, etc.) will be pooled together and sub-group analyses will be performed.

*Outcome measures*

- Odds ratio (OR) comparing the prevalence of soil-transmitted helminth infections among individuals having access to or using sanitation and those without will be used as effect measure. The OR will either be extracted from the published article or calculated by the reviewers (based on the prevalence rates of individuals having/using or not having/using sanitation). If the OR is not directly reported or cannot be readily extracted from the published data, the reviewers will contact the corresponding authors for additional information (e.g., data provided in 2x2 contingency tables). Studies with a smaller OR than 1.0 indicate a decrease in the odds of being infected with soil-transmitted helminths among those individuals having access or using sanitation.
- We aim at extracting information on both prevalence and intensity of soil-transmitted helminth infections. Should we identify at least 5 studies reporting data on infection intensity (measured in helminth eggs per gram of stool), we will perform separate meta-analyses.

*Publication type*

- Full published papers will be eligible (no language restrictions).
- Studies in which one or several of the authors have been involved with that are under peer-review at the time of search might be included.

**Search Methods**

We will search the following electronic databases:

- PubMed
- Embase
- ISI Web of Science
- WHO Library Database

There will be no restriction on language or year of publication.

The following keywords will be employed (see Table 1):

- “sanitation”
- “sanitary engineering”
- “water supply”
- “waste management”

combined with

- “helminth”
- “soil-transmitted helminth”
- “geohelminth”
- “ascaris”
- “lumbricoides”
- “trichuris”
- “trichiura”
- “hookworm”
- “ancylostoma”
- “duodenale”
- “necator”
- “americanus”

Additionally, bibliographies of identified publications and published reviews will be hand searched for potential additional relevant articles.

| **Sanitation** | **In combination with** | **Parasite** |
| --- | --- | --- |
| Sanitation, sanitary engineering, water supply, waste management |  | Helminth, soil-transmitted helminth, geohelminth, ascaris, lumbricoides, trichuris, trichiura, hookworm, ancylostoma, duodenale, necator, americanus |

*Table 1. Table visualizing the keywords of the electronic database search.*

**Data collection**

Two reviewers (KZ, BS) will independently scrutinize the list of titles, and if available the abstracts, to determine potential usefulness of the article. Final selection will be based on the full text of potentially relevant articles by the two reviewers independently. In cases of disagreement, a third reviewer (JK, JU) will examine such articles. Results will be discussed until reaching consensus among all three reviewers.

Relevant data, including a brief description of the study (e.g., study design, setting, year, sample size), the primary research question, details on the study population (e.g., all age groups, only children, only special groups) and the selection thereof (e.g., random selection), details on sanitation facilities (availability or use), and which soil-transmitted helminth species was investigated, will be extracted from all eligible studies by one reviewer into prepared data sheets and independently cross-checked by a second assessor.

**Quality assessment**

Critical quality appraisal of included studies will be conducted by the two reviewers independently based on a panel of criteria developed by the study group and which are based on the GRADE methodology (Atkins et al. 2004).

The quality assessment will be divided into three parts (Table 2). The first part will deal with a parasitological/diagnostic component. There will be up to three points for the diagnostic approach. One point will be assigned in the diagnostic test has been mentioned. A second point will be given if multiple stool samples or a suite of diagnostic tests have been employed. Finally, a third point will be given if a quality control scheme is in place (e.g., re-examination of a random sample of 10% of specimens). The second part of the quality assessment will cover the sanitation. One point can be gained if the toilet status (availability) was observed on the spot; no point if it was only assessed by a questionnaire. However, there will be a point for quality control, if the toilet status (e.g., cleanliness, suprastructure) was observed by repeated spot checks in a random sub-sample of the study population. In the third part, one point can be gained or lost if the study has other strengths or limitations that the reviewers feel are worth mentioning. In total, a study will be graded between -1 and +6 points.

Two reviewers (KZ, BS) will independently perform the quality assessment of each eligible study and document the results in separate tables. In a next step the results will be compared. If there are discrepancies, a third reviewer (JK or JU) will examine such articles, and results discussed until a consensus is found among the three assessors. No study will be excluded based on a low quality score.

| **Part** | **Feature** | **Points** |
| --- | --- | --- |
| Parasitology | Diagnostic approach | 0: not mentioned  1: diagnostic test mentioned  2: multiple tests/samples used |
|  | Quality control (parasitology) | 0: no or not mentioned  1: quality control performed |
| Sanitation | Toilet status assessed by | 0: questionnaire  1: team |
|  | Quality control (sanitation) | 0: no or not mentioned  1: quality control performed (team investigated toilet status) |
| Other quality measurement | Other limitations or strengths | -1: other limitations  +1: other strengths |

*Table 2: Table visualizing the quality control assessment.*

**Meta-analysis**

Separate meta-analyses will be performed for availability and use of sanitation facilities. Given the heterogeneity, analyses will further be divided into *A. lumbricoides*, *T. trichiura*, hookworm, and soil-transmitted helminths combined. Additionally, sub-group analyses will performed according to different age-groups and geographical areas.

Publication bias will be investigated using Egger’s test (Sterne et al. 2001). Moran’s *I^2^* and Cochrane’s *Q*-tests will be employed to determine the heterogeneity between studies. We define age of participants and type of sanitation as potential explanations of heterogeneity. In case of heterogeneity (*I^2^* > 50%), random effect models will be used throughout all analyses (DerSimonian and Laird 1986).

**References**

Asaolu SO, Ofoezie IE (2003) The role of health education and sanitation in the control of helminth infections. Acta Trop 86: 283-294.

Atkins D, Best D, Briss PA, Eccles M, Falck-Ytter Y, et al. (2004) Grading quality of evidence and strength of recommendations. BMJ 328: 1490.

DerSimonian R, Laird N (1986) Meta-analysis in clinical trials. Control Clin Trials 7: 177-188.

Esrey SA, Potash JB, Roberts L, Shiff C (1991) Effects of improved water supply and sanitation on ascariasis, diarrhoea, dracunculiasis, hookworm infection, schistosomiasis, and trachoma. Bull World Health Organ 69: 609-621.

Sterne JA, Egger M, Smith GD (2001) Systematic reviews in health care: investigating and dealing with publication and other biases in meta-analysis. BMJ 323: 101-105.
